# Clinical AI Scribes in primary care: accuracy, error severity and implications for clinical practice

**DOI:** 10.1136/bmjdhai-2025-000092

**Published:** 2025-09-28

**Authors:** Thomas C Draper, Timothy Cox, Kathryn Lamb-Riddell, Luigi Andrea Moretti, John McCormick, Stephen Trowell, Janice Kiely, Richard Luxton

**Affiliations:** 1 University of the West of England, Bristol, UK; 2 NHS England and NHS Improvement South West, Taunton, England, UK

**Keywords:** Documentation, Artificial intelligence, Informatics, Primary Health Care, Delivery of Health Care

## Abstract

**Objectives:**

To investigate the performance of commercially available Clinical Artificial Intelligence Scribes (CAISs), assessing their accuracy, potential clinical impact of errors, and documentation quality, given growing concerns around errors and safety.

**Methods and analysis:**

Seven CAIS products were investigated, using eight standardised clinical consultation scenarios recorded as audio. CAIS-generated summaries were assessed against a human-validated transcript and evaluated for errors (omissions, factual inaccuracies and hallucinations). Error severity was rated by medical doctors, generating a novel severity-weighted mpact Score (linear and exponential variants), to quantify potential clinical impact. Further analysis using the Physician Documentation Quality Instrument (PDQI-10) (a validated clinical note quality score) reinforced the findings.

**Results:**

Omissions dominated error counts (83.8%, p<<0.001), with CAISs varying widely in error frequency and severity, and a median of 1–6 omissions per consultation (depending on CAIS). Although less frequent, hallucinations and factual inaccuracies were more often clinically serious. No tested CAIS produced error-free summaries. The Impact Score highlighted clinical severity, notably amplifying the significance of less frequent but high-severity errors. PDQI-10 analysis indicated summaries were weakest in succinctness and organisation, but strong in consistency and clinical usefulness.

**Conclusions:**

The CAISs demonstrate high levels of summarisation accuracy. However, there is great disparity between the currently available CAIS products and, while some perform well, none are perfect. Clinicians should therefore maintain vigilance, particularly checking omitted psychosocial details and medications, and scrutinising plausible-sounding insertions. Purchasers and regulators should be aware of the significant performance disparities identified, reinforcing the need for careful evaluation and selection of CAIS products.

**WHAT IS ALREADY KNOWN ON THIS TOPIC**

- The use of Clinical Artificial Intelligence Scribes (CAISs) has proliferated in primary care settings, promising rapid consultation summarisation, but concerns about accuracy and clinical safety persist.

**WHAT THIS STUDY ADDS**

- A comparative assessment of seven commercially available CAISs across eight diverse consultation scenarios, systematically categorising summary errors and introducing a novel Impact Score to quantify their clinical severity.
- While omissions were most frequent, rare hallucinations and inaccuracies posed disproportionately high clinical impact.

**HOW THIS STUDY MIGHT AFFECT RESEARCH, PRACTICE OR POLICY**

- Clinicians should maintain careful vigilance when using CAISs, especially regarding omitted psychosocial details, medications and plausible-sounding inserted diagnoses or treatments.
- Purchasers and regulators must recognise substantial performance disparities between CAIS products, underlining the need for structured assessment and cautious implementation.

## Introduction

Since the popularisation of large language models (LLMs) by tools like ChatGPT,^1^ clinicians have increasingly incorporated them into daily practice; despite warnings, and in some cases suggested outright bans,^2^ from national and international organisations. The practical benefits offered by these artificial intelligence (AI) powered tools^3^ (including up to 70% administrative time saving^4^) often outweigh an individual’s concerns or regulatory caution. This trend underscores both the transformative potential of LLMs in healthcare and the urgent need to assess, regulate and define appropriate standards for their use.^5^

Recent reviews^6 7^ on the transformative potential of AI in healthcare have highlighted the potential advantages of the technology, together with risks and challenges, eg, data security, regulatory issues and acceptance by patients and clinicians.^8–10^ Recently, there has been a surge in the development of AI-powered scribe technologies which transcribe clinician-patient consultations into structured medical summaries,^11 12^ termed Clinical AI Scribes (CAISs). There are now a significant number of such packages available commercially. As of August 2025, Elion Health lists over 88 such products, having increased from 40 just 16 months previously.^13^

It has been proposed that the quality of the consultation is improved by these products, as the clinician does not need to make notes during the consultation, thereby improving patient engagement.^14^ The automated approach also has the potential to reduce the administrative burden on clinicians,^15^ thereby helping address burnout.^16–18^ It has also been reported^19^ that the AI scribe may reduce the risk of errors introduced by the clinician using manual input and increase the efficacy of electronic health record (EHR) management. Another avenue of exploration is the impact of CAISs on clinician workload and efficiency. While AI scribes offer the potential to reduce administrative burden, it remains to be seen whether their implementation leads to measurable improvements in workflow and patient outcomes.^9 20 21^

AI-generated summaries can be imperfect, and several techniques to determine and classify AI-generated errors exist.^12^ These include automated approaches, eg, ROUGE (Recall-Oriented Understudy for Gisting Evaluation) and BERT (Bidirectional Encoder Representations from Transformers),^22–25^ and also manual approaches^26–28^ in which the clinical notes are compared with the ground-truth original audio file. Several multidimensional metrics also exist, eg, a modified Physician Documentation Quality Instrument (PDQI-10)^29^ and the Sheffield Assessment Instrument for Letters.^30 31^ Asgari *et al* 32 included a risk assessment for each error, that is, combining clinical impact with the probability of occurrence.

The focus of this current paper is to (1) assess the quality of clinical summaries, (2) to quantify the type and number of errors within clinical summaries and (3) to assess the possible impact of these errors on the future health of the patient. We carried out such an assessment for seven commercial clinically-focussed AI scribes, purposely excluding non-medically focused AI scribes, eg, ChatGPT, which have previously been reported to introduce a significant number of errors.^26^

## Methodology

**Figure 1:**
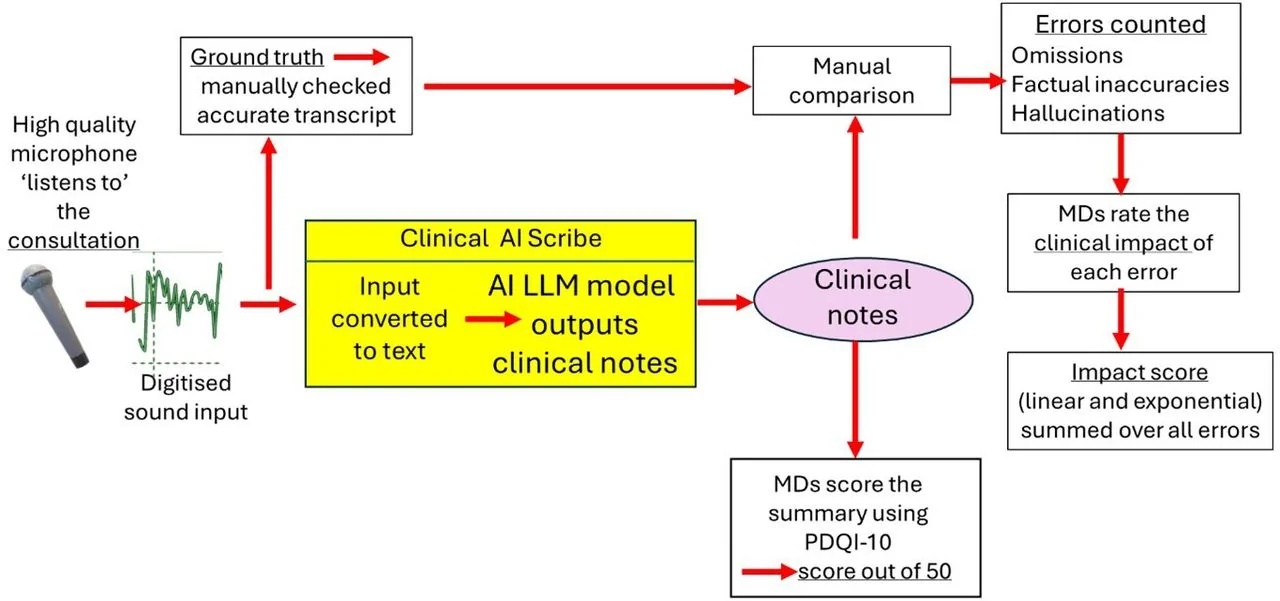
A graphical representation of the process for using a Clinical AI Scribe (CAIS), from the audio being collected by the microphone through processing by the CAIS, resulting in a clinical summary; along with the various evaluation methods. AI, artificial intelligence; LLM, large language model; MD, medical doctor; PDQI-10, Physician Documentation Quality Instrument.

All studies took place at the National Health Service (NHS) England South West Centre of Digital Excellence (CoDE) within the Health Technology Hub at the University of the West of England, Bristol, which includes a simulated primary care environment designed and approved by clinicians. This setup provided a realistic clinical environment, including arrangement of individuals and equipment, and acoustics. All medical consultations in this study were conducted with actors portraying simulated medical conditions. This section provides an overview (see figure 1); full details are provided in the online supplemental information.

10.1136/bmjdhai-2025-000092.supp1 Supplementary data

### Recording of simulated medical consultations

Simulated consultations were conducted by actors, rotating roles as clinician and patient, using standardised clinical scenarios: diarrhoea, sleep apnoea, prostate, panic attack, abdominal pain, headaches, skin rash and memory loss. Actors received a scenario brief outlining relevant clinical details; created with, and verified by, clinicians. Consultations had an average patient-facing duration of 7 min and 6 s. These recordings formed the foundation for subsequent experiments. Full scenario briefs are provided in the online supplemental information.

### Generation and analysis of the clinical summaries

Eight recorded consultations were provided as identical audio inputs to seven CAISs between August 2024 and January 2025. Default settings and a standard SOAP (Subjective, Objective, Assessment, Plan) template were used. For each consultation, CAIS-generated summaries were compared against a human-validated transcript, considered the gold standard for clinical content accuracy in this study. While transcripts and summaries differ in purpose, the transcript offered a consistent and comprehensive reference for evaluating clinical accuracy. Human-written summaries were not used due to variability and absence of a standardised reference format.

Each summary was analysed manually, by looking for omissions, hallucinations and factual inaccuracies:

- Omissions: Clinical details discussed in the consultation but missing from the summary. For example, failing to record nocturia.
- Hallucinations: Information in the summary not based on the original audio. For example, recording ‘no chest pain’ when chest pain was never discussed.
- Factual inaccuracies: Incorrectly reported details that contradict the original audio. For example, recording that symptoms have been ongoing for 1 week, rather than 1 year.

It was also noted whether the CAIS suggested either a differential diagnosis or specific tests. AI-powered diagnoses^33^ are not yet perfected,^34^ and there is an ongoing discussion as to their use.^35–39^

### Error severity weighting: the Impact Score

Identified errors were independently rated for clinical severity by four medical doctors (MDs) on a 1–5 scale (see table 1). These severity scores were averaged across reviewers to produce an error weighting ( w ).

**Table 1:** Definitions of the different weightings, and resulting Impact Scores, given to the CAIS-generated clinical errors. $I_{e}$ is taken to three significant figures

Weighting ( w )	Definition	$I_{l}$	$I_{e}$ $\left(e^{w}-e\right)$
1	Not relevant	1	0
2	Negligible *eg, recording a branded version of a drug instead of a generic one*.	2	4.67
3	Somehow relevant *eg, altering details which may be considered for a diagnosis/treatment*.	3	17.4
4	Very relevant *eg, altering details which play a crucial role in the clinical pathways, delaying diagnosis or impacting treatment*.	4	51.9
5	Fatal: *Generating a misunderstanding which leads to fatal consequences, either in emergency scenarios or in a domino effect in the long run*.	5	146

CAIS, Clinical Artificial Intelligence Scribe.

These weights were used in two ways. First, the Linear Impact Score ( $I_{l}=w$ ), which assigns equal weight per severity point, directly proportional to clinical severity. This straightforward approach provides a baseline severity-weighted measure of clinical error impact.

The Exponential Impact Score ( $I_{e}=e^{w}-e$ ) amplifies high-severity errors, reflecting the disproportionate clinical consequences of serious errors. This exponential scaling acknowledges that clinical severity increases non-linearly, with minor errors yielding negligible scores and very severe errors being heavily penalised (see table 1). Although exponential weighting is not commonly applied in medicine,^40^ medical adverse effects are known to escalate exponentially in severity,^41^ and exponential weighting has previously been used in both medical^42–44^ and non-medical^45 46^ error/risk analysis. The exponential Impact Score provides clinically meaningful differentiation by highlighting serious errors, even when their frequency is low.

Therefore, the Linear Impact Score ( $I_{l}=w$ ), assigns equal unit value per severity point; and the Exponential Impact Score ( $I_{e}=e^{w}-e$ ), amplifies the influence of high-severity errors. This reflects the assumption that a small number of serious clinical documentation errors may be more consequential than a larger number of minor ones.

Both Impact Scores are dimensionless metrics (with lower scores indicating better performance). Note that for a full risk assessment, this Impact Score would need to be modified (eg, multiplied) by the probability of occurrence.

### Physician Documentation Quality Instrument scoring

Each summary was independently evaluated by an MD using the PDQI-10,^29 47^ a validated measure adapted specifically for CAISs. PDQI-10 comprises 10 attributes (eg, accuracy, clarity, clinical usefulness), each rated on a 5-point Likert scale, for a maximum total of 50 points. Attributes are detailed in online supplemental table S1 (online supplemental information). Grading was performed by an MD while listening to the recordings, taking notes and referring to transcripts. PDQI-10 scoring provides a multidimensional assessment of documentation quality, complementing the Impact Score’s focus on error severity.

### Patient and public involvement statement

A Public/patient group (Citizens Reference Group) exists for the CoDE. They were consulted in the design phase of the study and were provided with early-stage results to which they gave feedback to further inform the study.

## Results

### Types and number of errors

**Figure 2:**
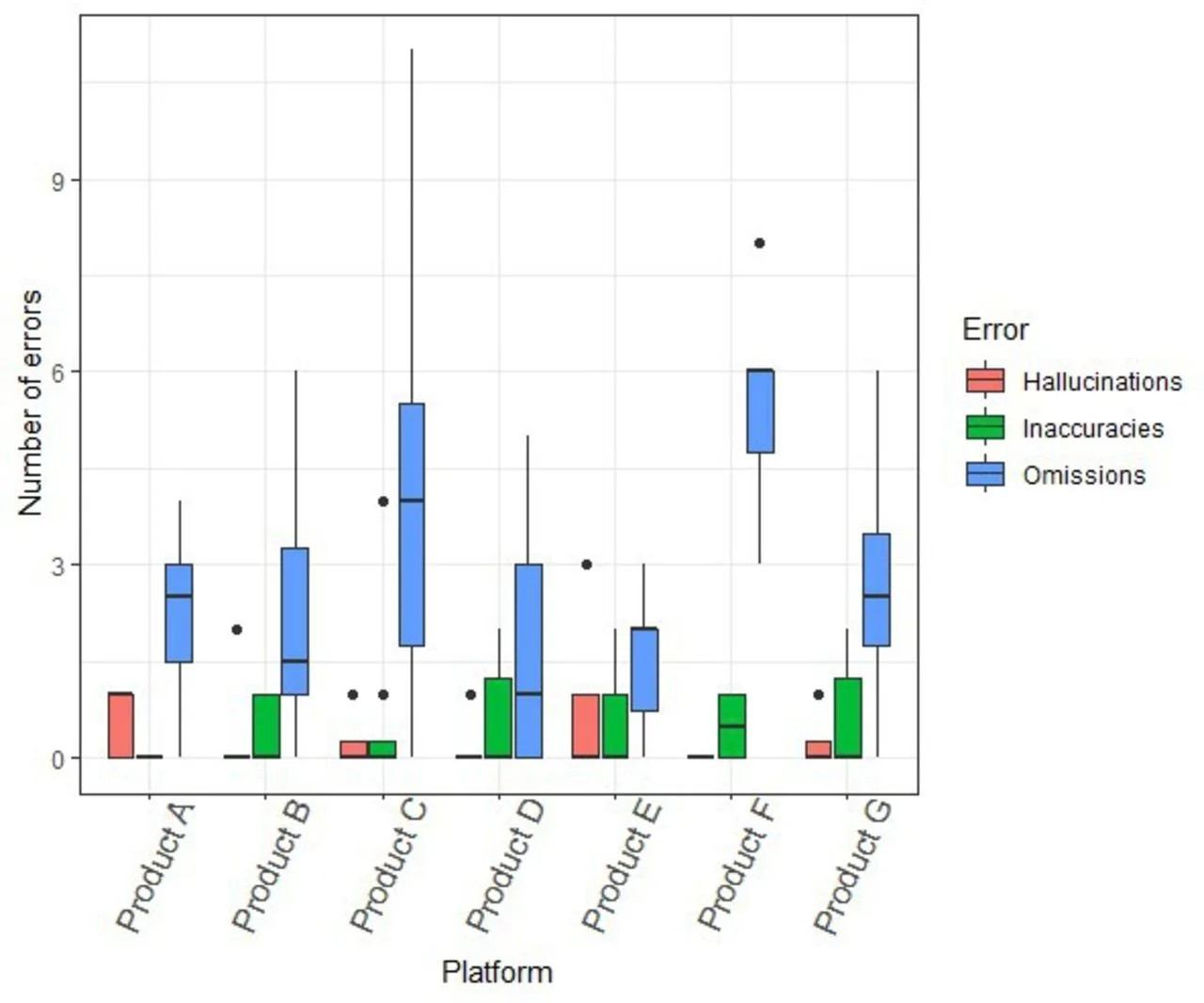
Number of errors per consultation across all scenarios, by CAIS and error type. Each boxplot shows the distribution of hallucinations, factual inaccuracies and omissions, averaged across scenarios for each product. Omissions were consistently the most common error type. Values can be seen in online supplemental table S2 in the online supplemental information. CAIS, Clinical Artificial Intelligence Scribe.

The number of errors observed in the generated clinical summaries varied considerably across CAIS systems, with some exhibiting a high frequency of errors while others demonstrated relatively fewer issues. This is clear from figure 2 and online supplemental table S2, where it can be seen that all of the CAISs produced imperfect summaries.

For all CAISs, the highest contributor to the total error count was omissions, significantly outnumbering other types (83.8% of all errors, one-way analysis of variance, p=0.00002), the median of which ranged from 1 to 6 per consultation for each CAIS. This suggests some CAISs are frequently excluding essential clinical information from the summary. Examples of omissions include missing details about family history, smoking habits and medication details.

Following omissions, factual inaccuracies constituted the second most common error type (8.3% of all errors). Most CAISs generated few factual inaccuracies (median ranging from 0 to 0.5 per consultation per CAIS); nevertheless, any factual inaccuracy indicates incorrect information present in the clinical summary. Examples included stating that the patient should contact a referred service (when the service would contact them), incorrect advice about alcohol consumption and recording pain in an incorrect anatomical location.

Hallucinations were the least frequently observed (7.8% of all errors, though not significantly different in number compared with factual inaccuracies, post-hoc Tukey's Honestly Significant Difference, p=0.959), but arguably the most concerning type of error due to their potential to introduce entirely false information in the patient’s EHR. The median number of hallucinations per consultation for each CAIS ranged from 0 to 1. Examples included assuming a (non-stated) medication, recording a non-smoker as a pipe smoker and inventing diagnoses.

While differences between CAISs were expected, it was interesting that their performance (error count) varied notably between different scenarios (see the heatmap in online supplemental figure S1, online supplemental information). Some scenarios had a much higher error count than others. For instance, the rash scenario had a median (IQR) error count of 5 (3–5), which was one of the worst performing scenarios. Conversely, the headache scenario had a median (IQR) error count of 0 (0–2), being the best performing via this metric (p=0.0065). This indicates that certain types of medical consultations may be more challenging for the CAIS to accurately summarise. Possible reasons for this include the variability in terminology used, the density of clinical information and different levels of diagnostic certainty. In the headache scenario, the clinician was clear with their diagnosis and treatment plan, whereas the rash consultation had uncertainty around the diagnosis, with further tests being required.

It was also noted whether each CAIS made unsolicited suggestions for either differential diagnoses or specific tests to be performed. None of the tested CAISs suggested any specific tests; however, four of them did suggest a diagnosis not proposed by the clinician (on a single occasion). For each CAIS, this diagnosis was during the ‘prostate’ scenario and was typically benign prostatic hyperplasia (not mentioned by clinician).

**Figure 3:**
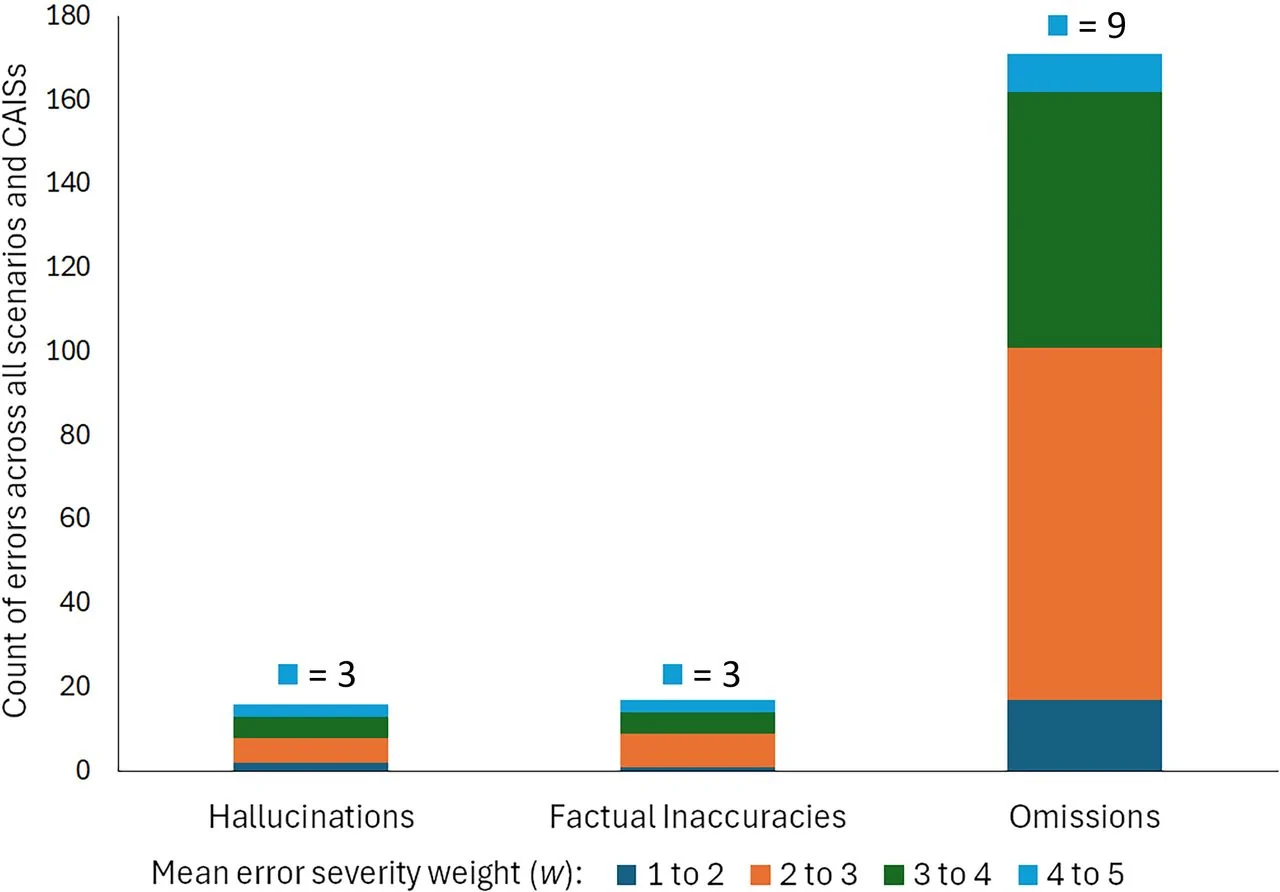
The total number of errors across all scenarios and CAISs, segmented by type and coloured according to their mean error severity weight ( w )*.* Numbers on top of the bars indicate the total number of that error type with w≥4 . Severity weight ≥4 includes scores rated as ‘very relevant’ (4) or ‘fatal’ (5). Full details can be found in online supplemental Table S3 *.* CAISs, Clinical Artificial Intelligence Scribes.

Across all CAISs and scenarios, hallucinations and factual inaccuracies were proportionally more likely to be severe, with 18.8% of hallucinations and 17.6% of factual inaccuracies assigned a severity weight of 4 or higher, compared with just 5.3% of omissions. However, due to their high frequency, omissions contributed the largest share of high-severity errors overall (60%). This is illustrated in figure 3, with full details provided in online supplemental table S3 (online supplemental information).

### Word counts

Analysis of AI-generated summary word counts revealed considerable variability between CAIS products, indicating differing approaches to summary style (online supplemental figure S2, online supplemental information). However, within a single CAIS, no significant differences were observed between scenarios (Kruskal-Wallis, p=0.98; see online supplemental figure S3, online supplemental information). The tight range within each CAIS, alongside the consistency across scenarios, suggests that summary length is primarily driven by product-specific design rather than scenario content or consultation duration. For example, although the ‘headache’ and ‘diarrhoea’ consultations varied substantially in length (around 5 min vs 11 min), the resulting summaries were of similar length. This implies that most CAISs aim for a fixed summary length, regardless of scenario complexity or consultation length.

We also examined whether longer summaries were associated with either fewer clinically significant errors (severity weight ≥4) or a lower total error count. Spearman correlation analyses showed weak associations (ρ=−0.18 and 0.12, respectively), neither of which was statistically significant (p=0.189 and p=0.389, respectively). This suggests that summary length alone does not reliably predict the risk of either severe errors or total error count.

### Impact Score

The errors generated by the seven CAISs were graded for clinical severity on a 5-point Likert scale by four MDs. The average severity score for each error ( w ) was used to calculate two forms of cumulative clinical impact: the Linear Impact Score ( $I_{l}=w$ ), which treats severity increases proportionally, and the Exponential Impact Score ( $I_{e}=e^{w}-e$ ), which gives disproportionately greater weight to high-severity errors. These scores reflect the overall clinical impact of the errors produced by each system, with lower values indicating fewer or less serious errors, while higher values may indicate a small number of high-impact mistakes or many low-impact ones.

**Figure 4:**
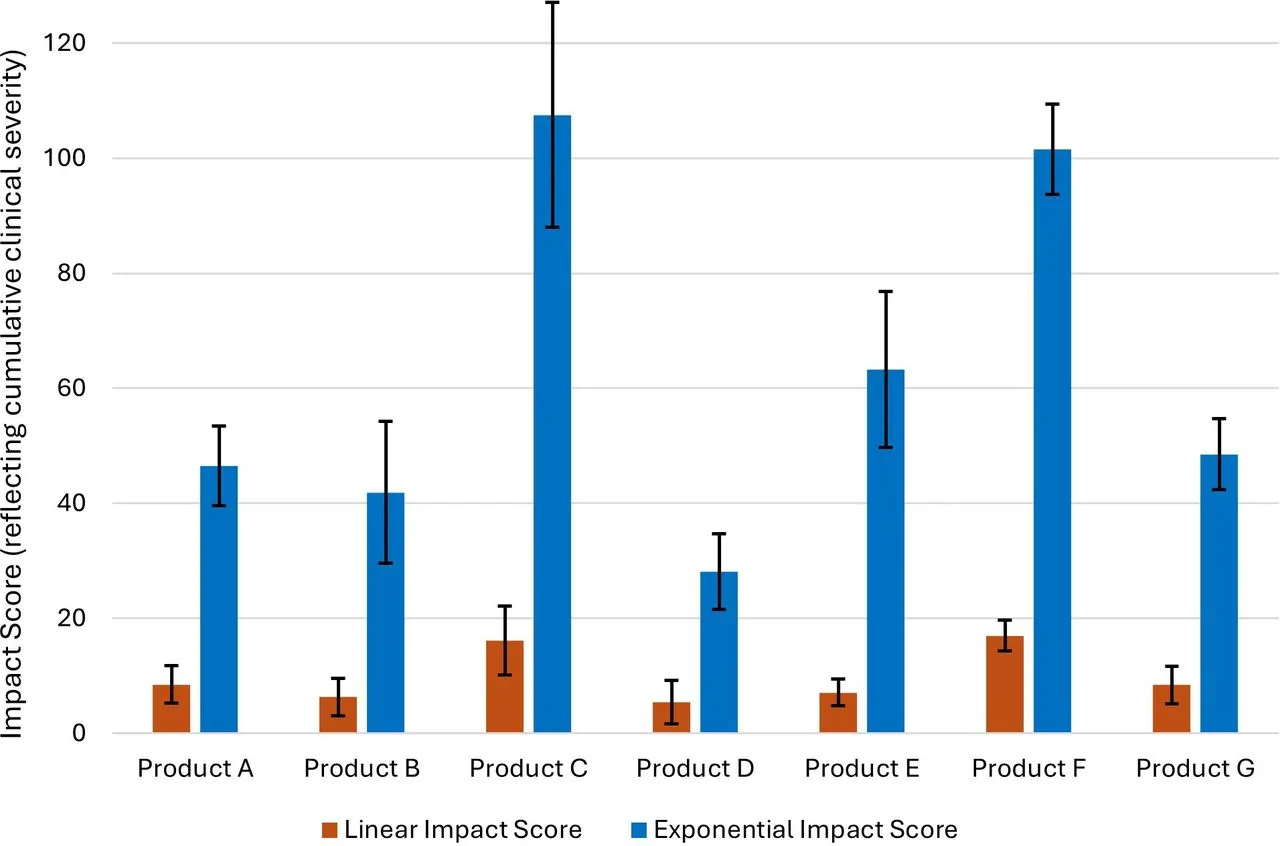
Cumulative clinical impact from summarisation errors across CAIS products, for all scenarios. The Linear Impact Score (orange) adds up severity weights for all identified errors, treating each unit of severity equally. The Exponential Impact Score (blue) amplifies the contribution of high-severity errors using a non-linear formula ( $I_{e}=e^{w}-e$ ), giving greater emphasis to potentially harmful mistakes. Thus, a product with only a few serious errors may score higher than one with many minor issues. Error bars indicate SE of the median, caused by variation across scenarios. Boxplots can be seen in online supplemental fi gure S6 (online supplemental information). CAISs, Clinical Artificial Intelligence Scribes.

Analysis of the Impact Scores (linear and exponential, figure 4) revealed notable performance variation across CAIS products. While several achieved low Impact Scores, performance often varied substantially depending on the specific clinical scenario. Most products demonstrated relatively consistent error severity, but typically showed notably poorer performance in at least one scenario, elevating their median Impact Score. Under the exponential scoring system, which emphasises high-severity errors, variability increased further, highlighting the disproportionate effect of even a small number of serious errors.

Looking closer at the scenario-based performance (see online supplemental figure S4-S6, online supplemental information) reveals that certain scenarios were consistently easier for all CAISs, whereas others presented substantial challenges. For instance, the ‘headache’ scenario appears to be relatively well-handled by most CAISs tried, as indicated by the low Impact Scores. This suggests that the ‘headache’ scenario may represent a simpler or more structured type of medical conversation that these CAISs are well-equipped to process accurately.

Conversely, the ‘sleep apnoea’ and ‘rash’ scenarios both presented significant difficulties, as evidenced by higher linear and exponential impact scores. The reason for this is unclear. However, potential explanations include the presence of complex speech patterns (eg, "No, not not really, no. It was, it, uh. My heart was really racing, but I didn’t. I wouldn’t say I had any, um, pain, really. Or, you know? Yeah. Shortness of breath*".*), ambiguous phrasing (eg, "no, no, no, no, yes") or uncertain diagnoses, all of which may challenge the AI models’ ability to generate accurate and complete summaries.

### PDQI scores

**Figure 5:**
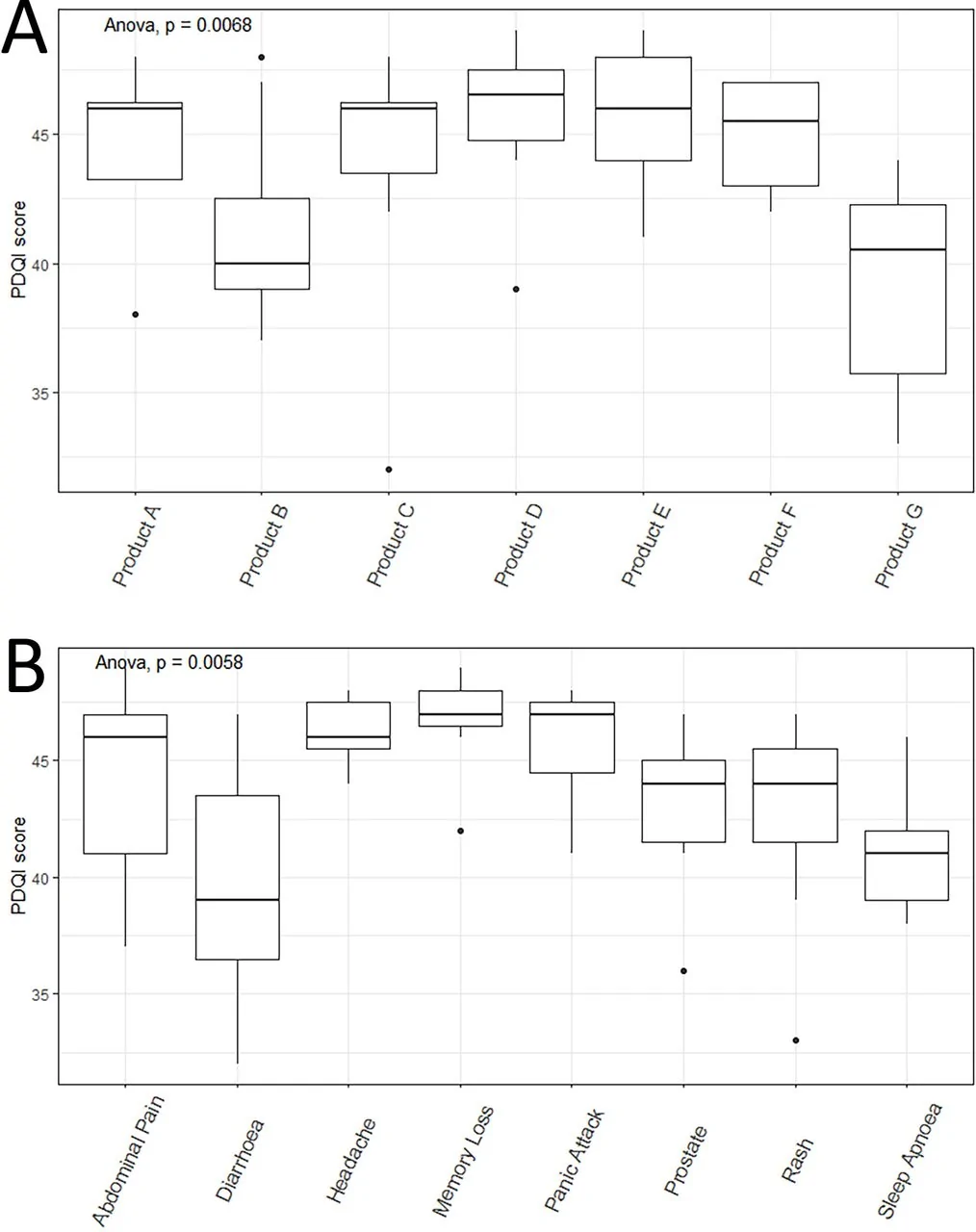
Variation in summary quality across scenarios and CAIS products, as a boxplot. (a) PDQI-10 scores (each out of a maximum of 50) assigned to each CAIS product across all scenarios, reflecting documentation quality across 10 domains, including accuracy, clarity, completeness and clinical usefulness. Higher scores indicate better quality. (b) The PDQI-10 scores for each scenario across all CAISs. Medians can be found in online supplemental table S2 (online supplemental information). ANOVA, analysis of variance; CAIS, Clinical Artificial Intelligence Scribe; PDQI, Physician Documentation Quality Instrument.

The quality of each CAIS-generated summary was evaluated using the PDQI-10, a validated 10-item measure of clinical documentation quality that assesses dimensions such as accuracy, organisation, succinctness and consistency.^29^ Each of the 10 attributes is scored on a five-point scale, yielding a maximum possible score of 50. The PDQI-10 scores for the different CAISs can be seen in figure 5(a); with a breakdown of their individual dimension scores shown in online supplemental figure S7, as a spider (radar) plot in online supplemental figure S8, and medians displayed in online supplemental table S2 (all in the online supplemental information).

Performance among the top CAIS products was notably close, with five out of seven achieving median PDQI-10 scores of at least 45.5, with the remaining two CAISs performing notably lower. Differences between CAISs were statistically significant (p=0.0068). Within each CAIS, there was also significant variability in scores across clinical scenarios (p=0.0058), with some scenarios generating large fluctuations in CAIS performance, while others produced more consistent scores across products (as seen by the larger boxes in figure 5b).

Certain clinical scenarios consistently yielded high or low PDQI-10 scores across most CAIS products. For example, the ‘memory loss’ scenario consistently demonstrated strong performance across all CAISs, whereas the ‘diarrhoea’ scenario typically yielded lower scores. This variability highlights the potential influence of scenario complexity and clinical context on CAIS documentation quality.

To better understand where CAISs tended to underperform, we analysed the distribution of PDQI-10 scores across the 10 documentation attributes. The lowest average scores were in ‘Succinct’ and ‘Organised’, suggesting summaries were often overly verbose or inconsistently structured. Conversely, the highest scores were seen in ‘Internally Consistent’, ‘Useful’ and ‘Comprehensible’, indicating strengths in coherence and overall clinical relevance. These patterns were generally consistent across scenarios and are illustrated in online supplemental figure S7 and S8 (online supplemental information). For example, one CAIS generated a 534-word summary for a skin-rash consultation, characterised by redundant phrases and repeated information, contributing to its low ‘Succinct’ score. Another CAIS exhibited significant organisational weaknesses, duplicating content in both a bullet list and SOAP-style note, repetitive mention of medication plans and a disjointed structure lacking logical flow. For example, the patient’s rash (suspected to be a side effect of apixaban, used for atrial fibrillation) was discussed separately and repetitively from the atrial fibrillation management itself, forcing clinicians to cross-reference sections and undermining narrative cohesion. These specific shortcomings contributed directly to a low score of 1 for ‘Organised’ (for this specific example).

## Discussion

### Interpretation of findings

Overall, these findings indicate that CAISs can demonstrate high levels of summarisation accuracy and quality. However, performance varies across CAIS systems, with notable disparities, particularly among the lower-performing models. They also reveal that the effectiveness of CAISs varies significantly depending on the scenario, with some models performing well in simple scenarios, but struggling in more ambiguous cases.

In addition to variation between products, several patterns of error recurred across multiple CAISs, suggesting common limitations in current-generation systems. Omissions were the most frequent and most widely shared, including failure to record psychosocial context (eg, *work-related stress,* or *concerns about dementia* or *cancer*), *medication effects* or *symptom specifics*. These issues appeared in more than half of the products and in some cases were assigned high ( w≥4 ) clinical severity. Representative examples of these, as well as hallucinations and factual inaccuracies, are provided in online supplemental table S4 in the online supplemental information.

Despite being (on average) individually rated as less severe, omissions were so frequent that they accounted for the majority of high-severity errors. While hallucinations and factual inaccuracies were more likely to receive high severity ratings, omissions made up: (1) 84% of all errors, and (2) 60% of errors individually rated as ‘very relevant’ or ‘fatal’. This reveals an important clinical insight: error frequency can amplify clinical impact. A large volume of omissions not only burdens the clinician with more corrections, but also increases the likelihood that at least one omission will carry substantial clinical consequences.

The Exponential Impact Score, which assigns greater emphasis to higher impact errors, underscores the importance of considering the severity of individual errors when evaluating CAIS performance. Notably, when error severity is accounted for, the performance differences between CAISs become less pronounced. These findings highlight that not all errors are equivalent in their clinical significance, and that treating them uniformly (or merely in ordinal terms) fails to adequately capture the nuances of CAIS performance.

Although the Impact Score provides a clinically meaningful view of error severity, particularly by amplifying the clinical implications of high severity ( w≥4 ) errors through its exponential weighting, we acknowledge that it is resource-intensive to apply, requiring manual clinician review and scoring of each error. While it offers valuable data for evaluation and audit purposes, it may not be practical for routine use in high-throughput environments. A more scalable variant (eg, using automated heuristics or sampling strategies, such as dip-sampling) may be developed in the future to approximate the same insights with reduced human effort.

The PDQI-10 ratings, reflecting overall clinical documentation quality, further reinforced that, despite several CAISs achieving high levels of summarisation accuracy, performance is not uniform across either CAISs or scenarios. While it was expected that some systems would perform better than others, it was surprising to observe that the scoring strongly correlated with the scenario. This reinforces that under ideal conditions, CAISs are able to produce clinical summaries which are of a sufficient standard; however, when the scenarios are more complex (eg, pre-existing conditions) or ambiguous (eg, unclear conversation or undiagnosed condition), the PDQI-10 scoring indicates that the generated summaries can be unsatisfactory.

It was interesting to observe that the PDQI-10 scores (reflecting broad documentation quality) only weakly correlated with the Impact Scores. This may reflect the broader scope of the PDQI, which includes 10 dimensions of documentation quality (see online supplemental table S1, online supplemental information), while the Impact Score isolates clinical impact severity. A stronger correlation was observed between the Impact Score and the isolated ‘Accuracy’ attribute of the PDQI-10 score, shown in online supplemental figure S9 (online supplemental information), with higher Accuracy scores associated with lower Impact Scores.

The PDQI-10 and the Impact Score serve complementary roles in the evaluation and deployment of CAISs. The PDQI-10, as a broad-based documentation quality tool, may be particularly useful for those conducting comparative assessments across products. Its multidimensional structure enables a holistic evaluation of clinical documentation quality. In contrast, the novel Impact Score offers a focused view of clinical impact, highlighting the potential severity of individual errors. This makes it a useful tool for internal audits, postmarket surveillance and safety reviews. We recommend that prospective users of CAISs consider using both metrics in tandem: PDQI-10 for general quality grading, and the Impact Score for evaluating and mitigating clinical impact. However, neither metric currently has a validated threshold for clinical acceptability,^29 47^ and we advise caution against interpreting either as a definitive sole marker of sufficiency.

### Implications for clinical practice

These findings have important implications for clinical practice. Minimising hallucinations, factual inaccuracies and omissions can enhance patient outcomes by ensuring that clinical records accurately reflect consultations. Conversely, a high omission rate may result in incomplete records, potentially compromising patient care and subsequent clinical decision-making. While hallucinations and factual inaccuracies occur less frequently, even a single error in these categories could have significant consequences, potentially necessitating additional human review and affecting workflow efficiency (or in a worst-case scenario, even resulting in an adverse effect).

Our results also suggest how clinicians might best use CAISs in practice. While a comprehensive review of all notes remains ideal, our study suggests that certain types of content warrant particular scrutiny. Psychosocial context (eg, stressors, concerns or social history), documentation of normal findings and patient-reported symptoms were the most frequently omitted, even in otherwise detailed summaries. Clinicians may therefore benefit from a more focused review strategy that prioritises these content domains. In addition, hallucinations, though less common, could involve inserted diagnoses or symptoms that were plausible but not actually discussed. Users should remain alert for such additions, especially in high-risk contexts.

It is important to remember that all of these products stipulate that a qualified clinician must review the generated summary before it is committed to the patient’s records. Consequently, a high number (if not all) of the errors should be eliminated, assuming that user overconfidence in the CAIS is avoided—an assumption which may not always be true. Even so, the total number of errors is still important, as it is known to directly correlate with the time necessary to correct the AI-generated summary.^48^ And as the time to check and correct errors increases, the utility of the CAIS decreases.

Our findings around the lower PDQI-10 scores in ‘Succinctness’ and ‘Organisation’ are particularly relevant for clinical usability. Overly verbose or poorly structured notes not only burden clinicians with additional cognitive load but may also lead to missed or misunderstood information. Product developers should consider prioritising improvements to these areas, to ensure clinical notes efficiently and effectively communicate patient information.

Beyond research settings, our evaluation framework may offer a practical foundation for the structured assessment of CAIS products. The combination of a transcript-based accuracy review, severity-weighted error analysis (the Impact Score) and a multidimensional quality score (the PDQI-10^29^) enables both granular and holistic appraisal. This approach could be adapted by healthcare systems to evaluate vendors, guide procurement or inform safety auditing. It may also provide product developers with clearer targets for performance optimisation.

Based on our findings, we propose the following practical considerations for clinicians using CAISs:

1. Be cautious of overconfidence: avoid assuming the AI is correct by default. Even when summaries appear well-structured, important omissions or hallucinations may still exist.
2. Review with focus: prioritise checking psychosocial history, normal findings and patient-reported symptoms. These domains were most commonly omitted.
3. Scrutinise for insertions: watch for inserted diagnoses or symptoms, particularly plausible-sounding additions that were not actually discussed during the consultation.
4. Do not equate length with quality: longer summaries were not necessarily more complete or accurate.
5. Be clear and structured: aim for a well-structured consultation, with any diagnosis or plan well defined.

### Limitations and future directions

While this study provides valuable insights, it is important to note some limitations. First, although the simulated consultations were conducted in a controlled environment designed and approved by NHS clinicians to closely replicate a real primary care environment, subtle nuances of real-world consultations (eg, interruptions, atypical patient behaviours) may not be fully captured. Therefore, our reported error rates may slightly underestimate those encountered with real patients in everyday clinical practice.

Second, the data set for this study is of finite scope and size. A larger, more diverse sample of medical consultations could enhance the robustness of the error analysis, provide more granular insights into the types of errors encountered and better capture the variability in clinical language and context.

Third, the assessment of errors relied on manual evaluation and the subjective judgements of clinical experts. Although the use of multiple clinicians and averaging of scores helped to mitigate individual bias, there remains an element of subjectivity in classifying errors.

It should also be noted that while performance varied across the CAISs we evaluated, the underlying causes of these differences remain unclear. Given the proprietary nature of these systems, our study is limited to describing observable outputs rather than attributing causality.

Future work should incorporate real clinical data from diverse healthcare settings to validate these findings under more variable conditions. Additionally, research on user interfaces and clinician interactions could ensure CAIS-generated summaries integrate smoothly into clinical workflows. Capturing transcript-level outputs may enable future assessment of word error rate and distinguish transcription from summarisation errors. Direct comparisons of CAIS-generated and clinician-written summaries would further contextualise AI performance. Finally, ongoing work must monitor ethical, legal and regulatory challenges to ensure safe and acceptable deployment of CAISs.

## Conclusions

In summary, our evaluation of commercial CAISs reveals significant differences in their performance, particularly regarding clinically relevant errors such as hallucinations, omissions and factual inaccuracies. The use of the established PDQI-10 instrument alongside our novel, severity-weighted Impact Score provided complementary insights into the overall quality and clinical implications of these errors. These findings emphasise the necessity for ongoing refinement of CAISs, as well the importance of critical scrutiny by clinicians, regulators and healthcare purchasers.

At the current stage, practical barriers to widespread adoption remain primarily ethical, legal and regulatory rather than technological. However, as CAISs increasingly integrate into clinical workflows, these challenges must be proactively addressed to ensure safe and effective deployment.

## Data Availability

Data are available upon reasonable request. Outputs from the commercial Clinical AI Scribes, transcripts of the original audio files, error counts and PDQI scoring will be made available upon reasonable request to the corresponding author. Structured descriptions of the eight clinical scenarios can be found in the supplementary information.

## References

[1] ChatGPT 4o. 2024. Available: https://chatgpt.com/

[2] Taylor J . AMA calls for stronger ai regulations after doctors use chatgpt to write medical notes. The Guardian; 2023.

[3] Liu J , Wang C , Liu S . Utility of ChatGPT in Clinical Practice. J Med Internet Res 2023;25:e48568. 10.2196/48568 37379067 PMC10365580

[4] Tangsrivimol JA , Darzidehkalani E , Virk HUH , et al . Benefits, limits, and risks of ChatGPT in medicine. Front Artif Intell 2025;8:1518049. 10.3389/frai.2025.1518049 39949509 PMC11821943

[5] Wang C , Liu S , Yang H , et al . Ethical Considerations of Using ChatGPT in Health Care. J Med Internet Res 2023;25:e48009. 10.2196/48009 37566454 PMC10457697

[6] Chustecki M . Benefits and Risks of AI in Health Care: Narrative Review. Interact J Med Res 2024;13:e53616. 10.2196/53616 39556817 PMC11612599

[7] Al Kuwaiti A , Nazer K , Al-Reedy A , et al . A Review of the Role of Artificial Intelligence in Healthcare. J Pers Med 2023;13:951. 10.3390/jpm13060951 37373940 PMC10301994

[8] Rajpurkar P , Chen E , Banerjee O , et al . AI in health and medicine. Nat Med 2022;28:31–8. 10.1038/s41591-021-01614-0 35058619

[9] van Buchem MM , Kant IMJ , King L , et al . Impact of a Digital Scribe System on Clinical Documentation Time and Quality: Usability Study. JMIR AI 2024;3:e60020. 10.2196/60020 39312397 PMC11459111

[10] Gebauer S , Eckert C . Survey of US physicians’ attitudes and knowledge of AI. BMJ Evid Based Med 2024;29:279–81. 10.1136/bmjebm-2023-112726 38355284

[11] van Zandvoort D , Wiersema L , Huibers T , et al . Enhancing summarization performance through transformer-based prompt engineering in automated medical reporting. 17th International Conference on Health Informatics; Rome, Italy, 2024:154–65. 10.5220/0012422600003657

[12] Gebauer S . Benchmarking and datasets for ambient clinical documentation: a scoping review of existing frameworks and metrics for ai-assisted medical note generation. medRxiv [Preprint] 2025. 10.1101/2025.01.29.25320859

[13] Elion - ai ambient scribe products. 2025. Available: https://elion.health/categories/ai-ambient-scribes/products

[14] Asan O , Young HN , Chewning B , et al . How physician electronic health record screen sharing affects patient and doctor non-verbal communication in primary care. Patient Educ Couns 2015;98:310–6. 10.1016/j.pec.2014.11.024 25534022 PMC4319541

[15] Seth P , Carretas R , Rudzicz F . The Utility and Implications of Ambient Scribes in Primary Care. JMIR AI 2024;3:e57673. 10.2196/57673 39365655 PMC11489790

[16] Mess SA , Mackey AJ , Yarowsky DE . Artificial Intelligence Scribe and Large Language Model Technology in Healthcare Documentation: Advantages, Limitations, and Recommendations. Plast Reconstr Surg Glob Open 2025;13:e6450. 10.1097/GOX.0000000000006450 39823022 PMC11737491

[17] Duggan MJ , Gervase J , Schoenbaum A , et al . Clinician Experiences With Ambient Scribe Technology to Assist With Documentation Burden and Efficiency. JAMA Netw Open 2025;8:e2460637. 10.1001/jamanetworkopen.2024.60637 39969880 PMC11840636

[18] Wu Y , Wu M , Wang C , et al . Evaluating the Prevalence of Burnout Among Health Care Professionals Related to Electronic Health Record Use: Systematic Review and Meta-Analysis. JMIR Med Inform 2024;12:e54811. 10.2196/54811 38865188 PMC11208837

[19] Van Veen D , Van Uden C , Blankemeier L , et al . Adapted large language models can outperform medical experts in clinical text summarization. Nat Med 2024;30:1134–42. 10.1038/s41591-024-02855-5 38413730 PMC11479659

[20] Peterson Health Technology Institute . Adoption of artificial intelligence in healthcare delivery systems: early applications and impacts. 2025.

[21] Haberle T , Cleveland C , Snow GL , et al . The impact of nuance DAX ambient listening AI documentation: a cohort study. J Am Med Inform Assoc 2024;31:975–9. 10.1093/jamia/ocae022 38345343 PMC10990544

[22] Yim W-W , Fu Y , Ben Abacha A , et al . Aci-bench: a Novel Ambient Clinical Intelligence Dataset for Benchmarking Automatic Visit Note Generation. Sci Data 2023;10:586. 10.1038/s41597-023-02487-3 37673893 PMC10482860

[23] Pyne Y , Wong YM , Fang H , et al . Analysis of ‘One in a Million’ primary care consultation conversations using natural language processing. BMJ Health Care Inform 2023;30:e100659. 10.1136/bmjhci-2022-100659 PMC10151863 37116948

[24] Zhang Y , Merck D , Tsai E , et al . Optimizing the factual correctness of a summary: a study of summarizing radiology reports. Proceedings of the 58th Annual Meeting of the Association for Computational Linguistics; 2020:5108–20. 10.18653/v1/2020.acl-main.458

[25] Alsentzer E , Murphy J , Boag W , et al . Publicly available clinical bert embeddings. Proceedings of the 2nd Clinical Natural Language Processing Workshop; 2019:72–8. 10.18653/v1/W19-1909

[26] Kernberg A , Gold JA , Mohan V . Using ChatGPT-4 to Create Structured Medical Notes From Audio Recordings of Physician-Patient Encounters: Comparative Study. J Med Internet Res 2024;26:e54419. 10.2196/54419 38648636 PMC11074889

[27] Draper TC , Leake J , Lamb-Riddell K , et al . Impact of acoustic and informational noise on AI-generated clinical summaries. BMJ Digit Health 2025;1:e000057. 10.1136/bmjdhai-2025-000057 PMC13528354 42712284

[28] Quiroz JC , Laranjo L , Kocaballi AB , et al . Challenges of developing a digital scribe to reduce clinical documentation burden. NPJ Digit Med 2019;2:114. 10.1038/s41746-019-0190-1 31799422 PMC6874666

[29] Tierney AA , Gayre G , Hoberman B , et al . Ambient Artificial Intelligence Scribes to Alleviate the Burden of Clinical Documentation. NEJM Catalyst 2024;5:1–15. 10.1056/CAT.23.0404

[30] Crossley GM , Howe A , Newble D , et al . Sheffield Assessment Instrument for Letters (SAIL): performance assessment using outpatient letters. Med Educ 2001;35:1115–24. 10.1046/j.1365-2923.2001.01065.x 11895235

[31] Balloch J , Sridharan S , Oldham G , et al . Use of an ambient artificial intelligence tool to improve quality of clinical documentation. Future Healthc J 2024;11:100157. 10.1016/j.fhj.2024.100157 39371531 PMC11452835

[32] Asgari E , Montaña-Brown N , Dubois M , et al . A framework to assess clinical safety and hallucination rates of LLMs for medical text summarisation. NPJ Digit Med 2025;8:274. 10.1038/s41746-025-01670-7 40360677 PMC12075489

[33] Zeltzer D , Herzog L , Pickman Y , et al . Diagnostic Accuracy of Artificial Intelligence in Virtual Primary Care. Mayo Clin Proc Digit Health 2023;1:480–9. 10.1016/j.mcpdig.2023.08.002 40206313 PMC11975709

[34] Jin Q , Chen F , Zhou Y , et al . Hidden flaws behind expert-level accuracy of multimodal GPT-4 vision in medicine. NPJ Digit Med 2024;7:190. 10.1038/s41746-024-01185-7 39043988 PMC11266508

[35] Prokopowicz D . Opportunities and threats to the development of artificial intelligence applications and the need for normative regulation of this development. IJOLS 2023;14:95–129. 10.5604/01.3001.0054.2699

[36] Pantanowitz L , Hanna M , Pantanowitz J , et al . Regulatory Aspects of Artificial Intelligence and Machine Learning. Mod Pathol 2024;37:100609. 10.1016/j.modpat.2024.100609 39260776

[37] Blumenthal D , Patel B . The Regulation of Clinical Artificial Intelligence. NEJM AI 2024;1:1–6. 10.1056/AIpc2400545

[38] Mennella C , Maniscalco U , De Pietro G , et al . Ethical and regulatory challenges of AI technologies in healthcare: A narrative review. Heliyon 2024;10:e26297. 10.1016/j.heliyon.2024.e26297 38384518 PMC10879008

[39] Weissman GE . Evaluation and Regulation of Artificial Intelligence Medical Devices for Clinical Decision Support. Annu Rev Biomed Data Sci 2025;8:81–99. 10.1146/annurev-biodatasci-103123-095824 39971383 PMC12339208

[40] Kohn LT , Corrigan JM , Donaldson MS . To Err Is Human: Building a Safer Health System. Washington, DC: Institute of Medicine, 2000. 25077248

[41] Bohl DD , Ahn J , Lukasiewicz AM , et al . Severity Weighting of Postoperative Adverse Events in Orthopedic Surgery. Am J Orthop (Belle Mead NJ) 2017;46:E235–43. 28856354

[42] Michels K , Song M , Willett WC , et al . Latency estimation for chronic disease risk: a damped exponential weighting model. Eur J Epidemiol 2020;35:807–19. 10.1007/s10654-020-00658-9 32728914 PMC7530062

[43] Mihaylova B , Briggs A , O’Hagan A , et al . Review of statistical methods for analysing healthcare resources and costs. Health Econ 2011;20:897–916. 10.1002/hec.1653 20799344 PMC3470917

[44] Manning WG , Basu A , Mullahy J . Generalized modeling approaches to risk adjustment of skewed outcomes data. J Health Econ 2005;24:465–88. 10.1016/j.jhealeco.2004.09.011 15811539

[45] Chang K-H , Chang Y-C , Lai P-T . Applying the concept of exponential approach to enhance the assessment capability of FMEA. J Intell Manuf 2014;25:1413–27. 10.1007/s10845-013-0747-9

[46] Leveson NG . Engineering a Safer World. Cambridge, MA: The MIT Press, 2012.

[47] Stetson PD , Bakken S , Wrenn JO , et al . Assessing Electronic Note Quality Using the Physician Documentation Quality Instrument (PDQI-9). Appl Clin Inform 2012;3:164–74. 10.4338/aci-2011-11-ra-0070 22577483 PMC3347480

[48] Moramarco F , Papadopoulos Korfiatis A , Perera M , et al . Human evaluation and correlation with automatic metrics in consultation note generation. Proceedings of the 60th Annual Meeting of the Association for Computational Linguistics (Volume 1:Long Papers); Dublin, Ireland, 2022:5739–54. 10.18653/v1/2022.acl-long.394

