# Supplementary material for: Clinical AI Scribes in primary care: accuracy, error severity and implications for clinical practice
Source: BMJ Digit Health Ai. 2025 Sep 28;1(1):e000092. doi: 10.1136/bmjdhai-2025-000092 (PMC13528381; doi:10.1136/bmjdhai-2025-000092)
Supplement: Supplementary file 1 [file bmjdhai-1-1-bmjdhai-2025-000092-s001.pdf]

Clinical AI Scribes in Primary Care: Accuracy, Error Severity, and Implications for Clinical Practice

Thomas C. Draper, [a]* Timothy Cox, [a] Kathryn Lamb-Riddell, [a] Luigi Andrea Moretti, [a] John McCormick, [b] Stephen Trowell, [b] Janice Kiely, [a] Richard Luxton [a]

(a) Centre of Digital Excellence, University of the West of England, Bristol, BS16 1QY, UK
(b) NHS England South West, South West House, Blackbrook Ave, Taunton, TA1 2PX, UK

* Corresponding author: tom.draper@uwe.ac.uk

Supplementary Information

Expanded Method – Additional Details

Recording of Simulated Medical Consultations

The simulations took place with six actors rotating between roles as either the clinician (as a General Practitioner) or the patient. Each consultation was recorded using two video cameras, positioned to capture the front of each actor, and a single dedicated microphone (Fifine K668) positioned midway between the clinician and the patient. Care was made to minimise any background noise. Prior to each consultation, actors received a scenario brief outlining relevant clinical details; these were created with, and verified by, clinicians. The actors were medically aware with respect to their assigned scenarios.

Generation and Analysis of the Clinical Summaries

In order to provide for a fair comparison, the same audio file was presented to the seven different AI packages. This was achieved either through a direct upload of the audio file, or by playing the audio file and re-routing the computer's input to its own stereo mix output using software (i.e. without cables, thereby eliminating any possible sound quality degradation). All experiments were conducted between August 2024 and January 2025. Each of the seven AI packages generated a unique clinical summary for each of the eight consultations. In all cases, the default settings of each individual AI package were used, along with the SOAP (Subjective, Objective, Assessment, Plan) template.

For each of the eight consultations an accurate human-verified transcript was produced. This transcript was considered the gold standard, because all of the medical information in the transcript should be present in the clinical summary; and, equally, all of the summarised clinical information should have been originally present at the consultation. While transcripts and summaries differ in purpose, the transcript offered a consistent and comprehensive

Draper TC, *et al. BMJ Digit Health* 2025; 1:e000092. doi: 10.1136/bmjdhai-2025-000092

reference for evaluating clinical accuracy. Only omissions of clinically meaningful content were counted as errors. Human-written summaries were not used due to variability and absence of a standardised reference format.

Each of the 56 generated summaries were then analysed manually, by looking for omissions, hallucinations, and factual inaccuracies:

- Omissions: Key pieces of clinical information that were not included in the summary, but were present in the original audio input. E.g. failing to record nocturia.
- Hallucinations: Instances where the CAIS tool generated content that was not based on the original audio input, causing *false* information to be included in the summary. E.g. recording "no chest pain", when chest pain was never discussed.
- Factual Inaccuracies: Errors where the tool's summary did not accurately reflect the spoken facts, causing *false* information to be included in the summary. E.g. recording that symptoms have been ongoing for 1 week, rather than 1 year.

In addition, it was noted whether the AI package, of its own volition, suggested either a differential diagnosis or specific tests.

Statistical Analysis

Statistical analyses, using nonparametric tests, were conducted to evaluate the significance of observed variations. Statistical analysis was conducted using either R in R Studio [1,2] or SPSS Statistics [3]. Nonparametric analysis was used to compare number of errors and error rates, employing the Exact two-sample Kolmogorov-Smirnov test and Mann-Whitney U 2 tailed test and Wilcoxon rank sum test with continuity correction. One-way Analysis of Variance (ANOVA) was used to compare between groups. Spearman's rank correlation was used to test for associations between word count and errors. Medians are reported along with the interquartile range (IQR) in brackets immediately following. Significance is taken as $p < 0.05$.

Draper TC, *et al. BMJ Digit Health* 2025; 1:e000092. doi: 10.1136/bmjdhai-2025-000092

Figures

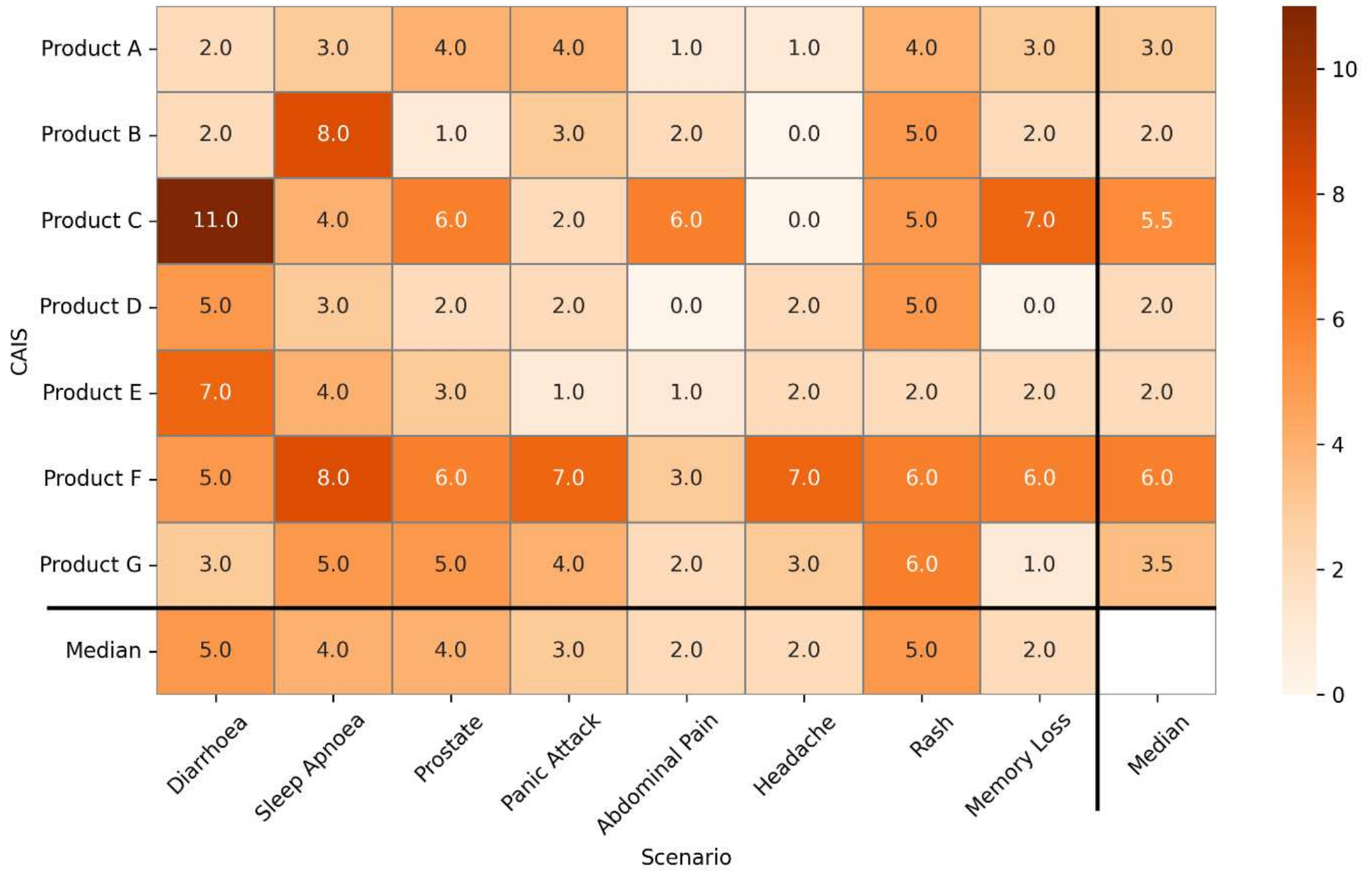

Figure S1: *A heatmap showing the number of total errors per CAIS and scenario type. Medians are given per CAIS and per scenario.*

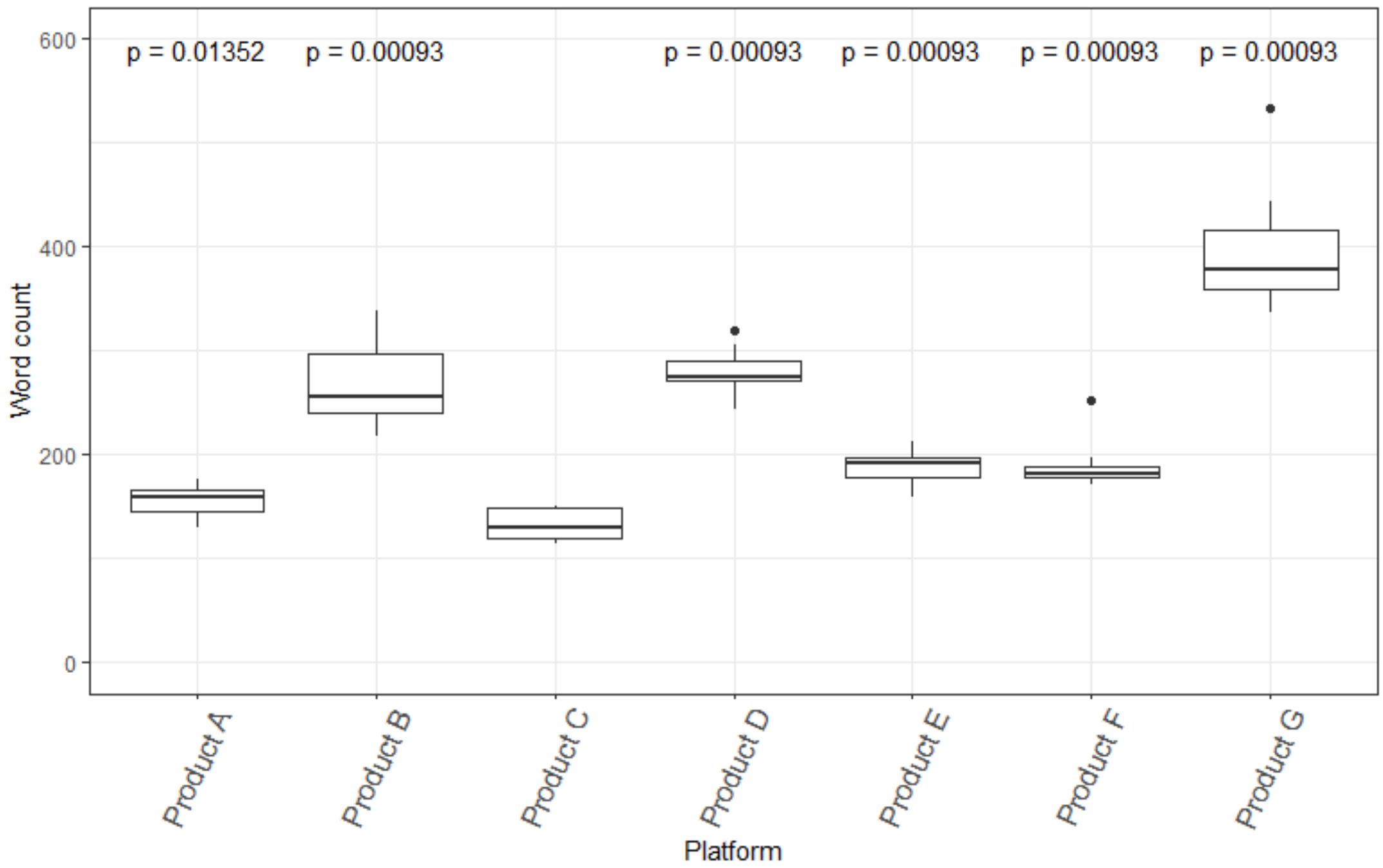

Figure S2: *Variation in summary length between CAIS products. A box-and-whisker plot showing the total number of words in the summaries generated by each AI product, pooled across all clinical scenarios. Each box represents the distribution of word counts for a single product, with lines and dots indicating range and outliers. Statistical comparisons were performed against the shortest summaries overall (Product C) using the Wilcoxon signed-rank test (p-values shown at top of plot).*

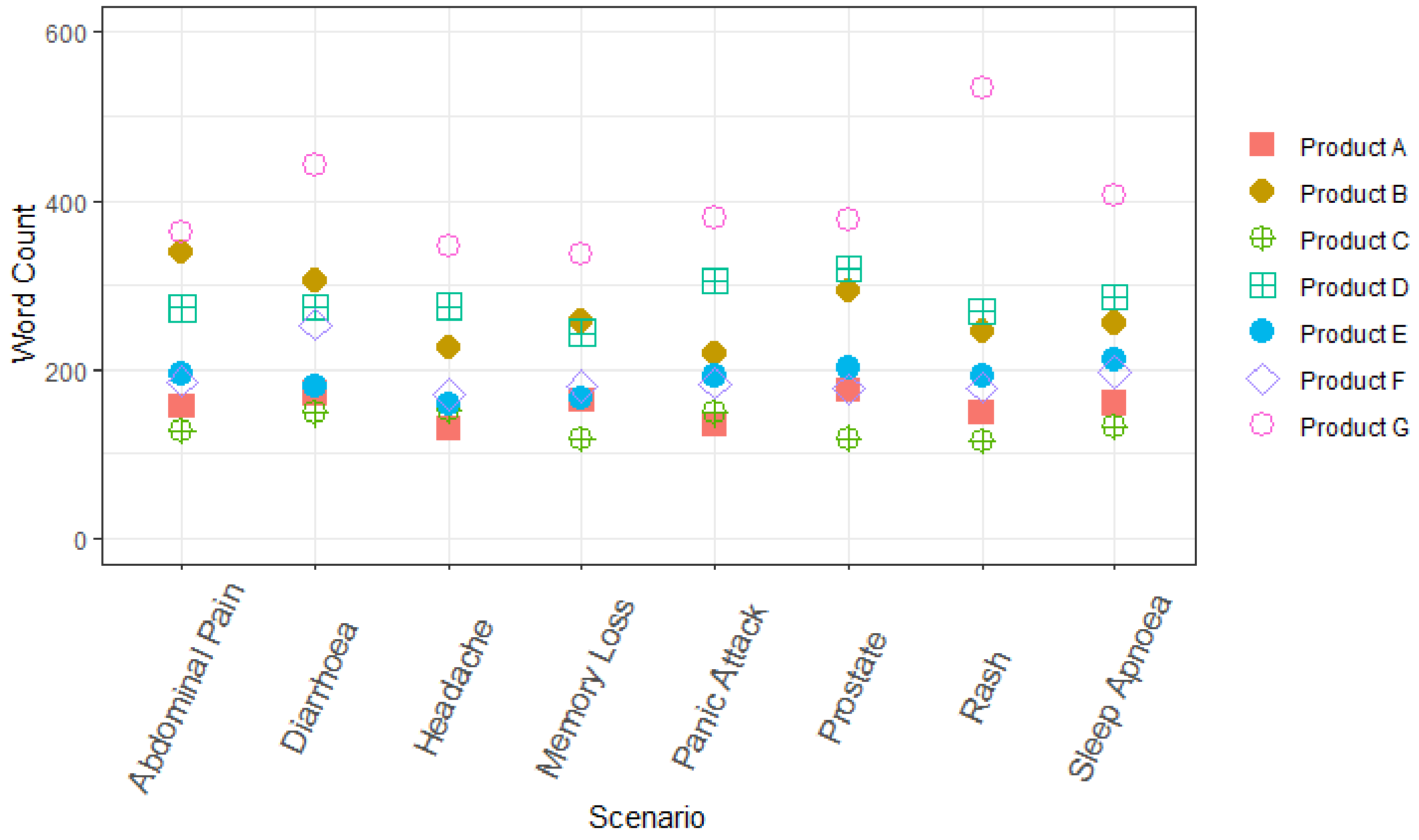

Figure S3: *Word count of CAIS generated summaries by clinical scenario. Each point shows the number of words in the summary generated by a single AI scribe for a given consultation type. Data shown for all seven products across eight clinical scenarios. Specifics can be seen in table S2.*

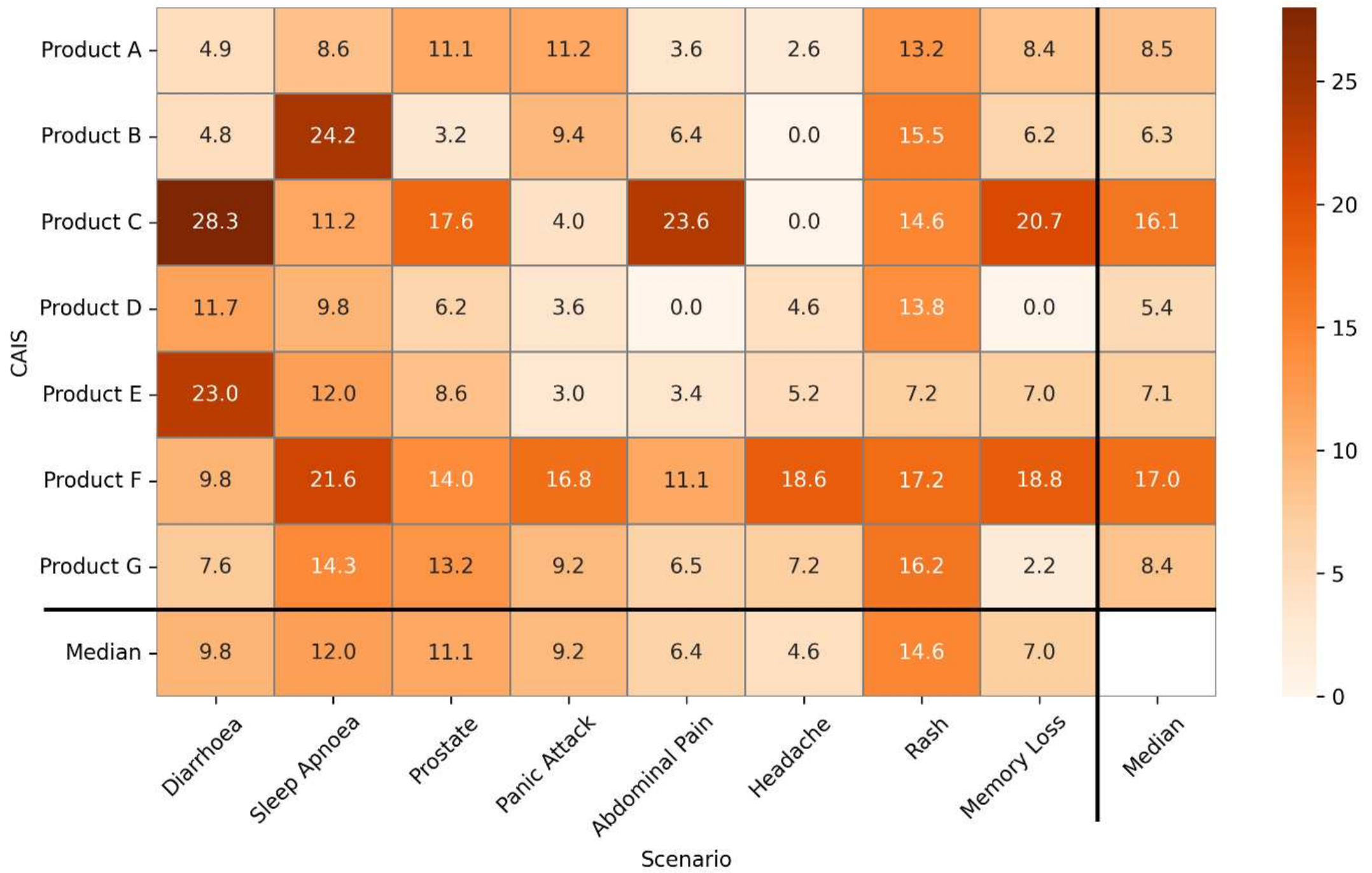

Figure S4: *The linear Impact Score for all eight scenarios and seven CAISs. Linear Impact Scores are calculated by* $I_l = w$*, where* I_l *is the Impact Score and* w *is the weight.*

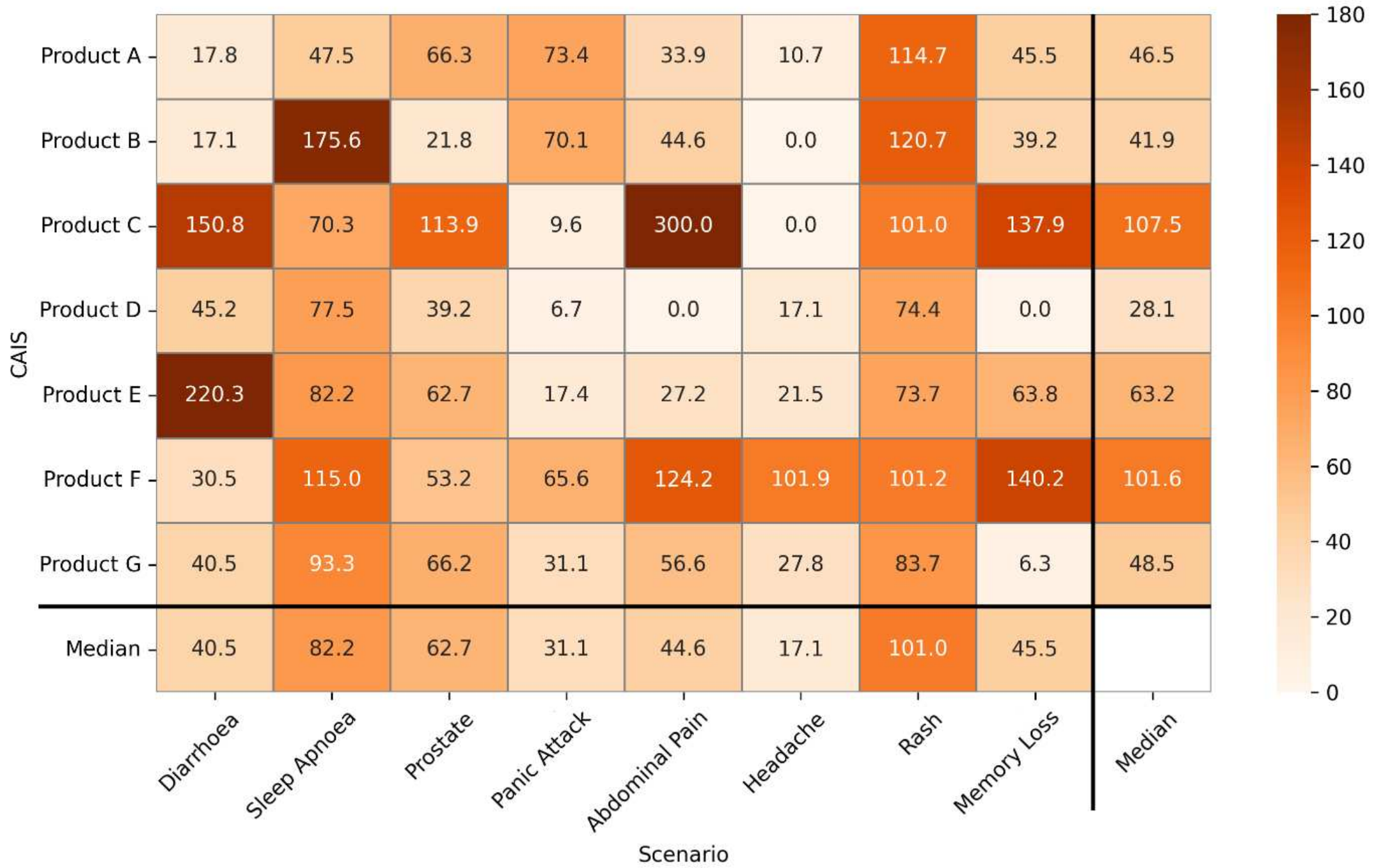

Figure S5: *The exponential Impact Score for all eight scenarios and seven CAISs. Exponential Impact Scores are calculated by* $I_e = e^w - e$*, where* I_e *is the Impact Score and* w *is the weight.*

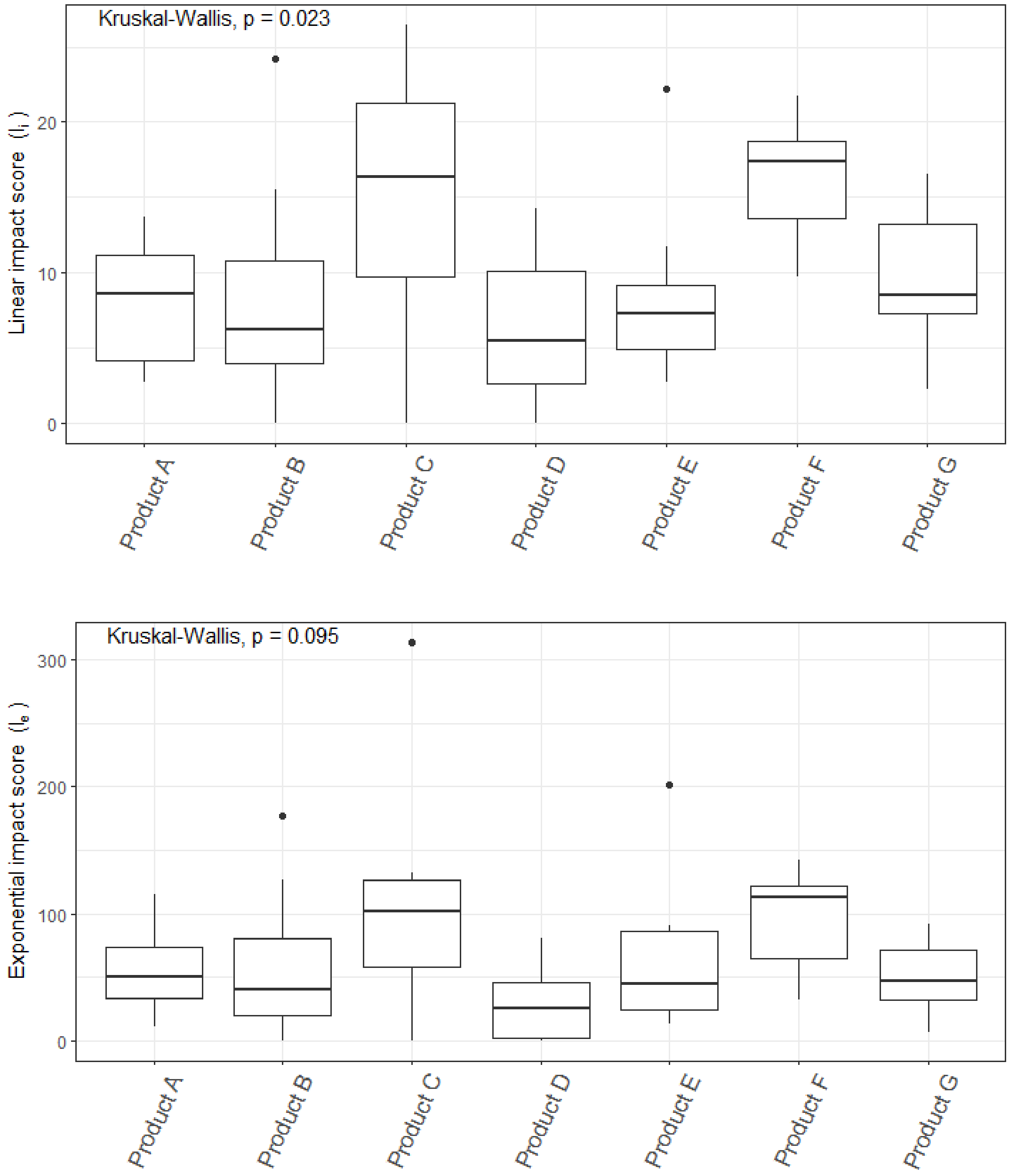

Figure S6: *Box plots showing (a) Linear Impact Scores, calculated by summing the severity weights of errors ($I_l = w$), and (b) Exponential Impact Scores, which emphasise high-severity errors ($I_e = e^w - e$). Higher scores indicate a greater cumulative clinical risk. Plots include scores from all seven CAIS products across eight clinical scenarios. Result of a Kruskal-Wallis statistical test (comparing variation between CAISs) are shown. Equivalent heatmaps and specifics can be seen in figures S4 and S5, and table S2.*

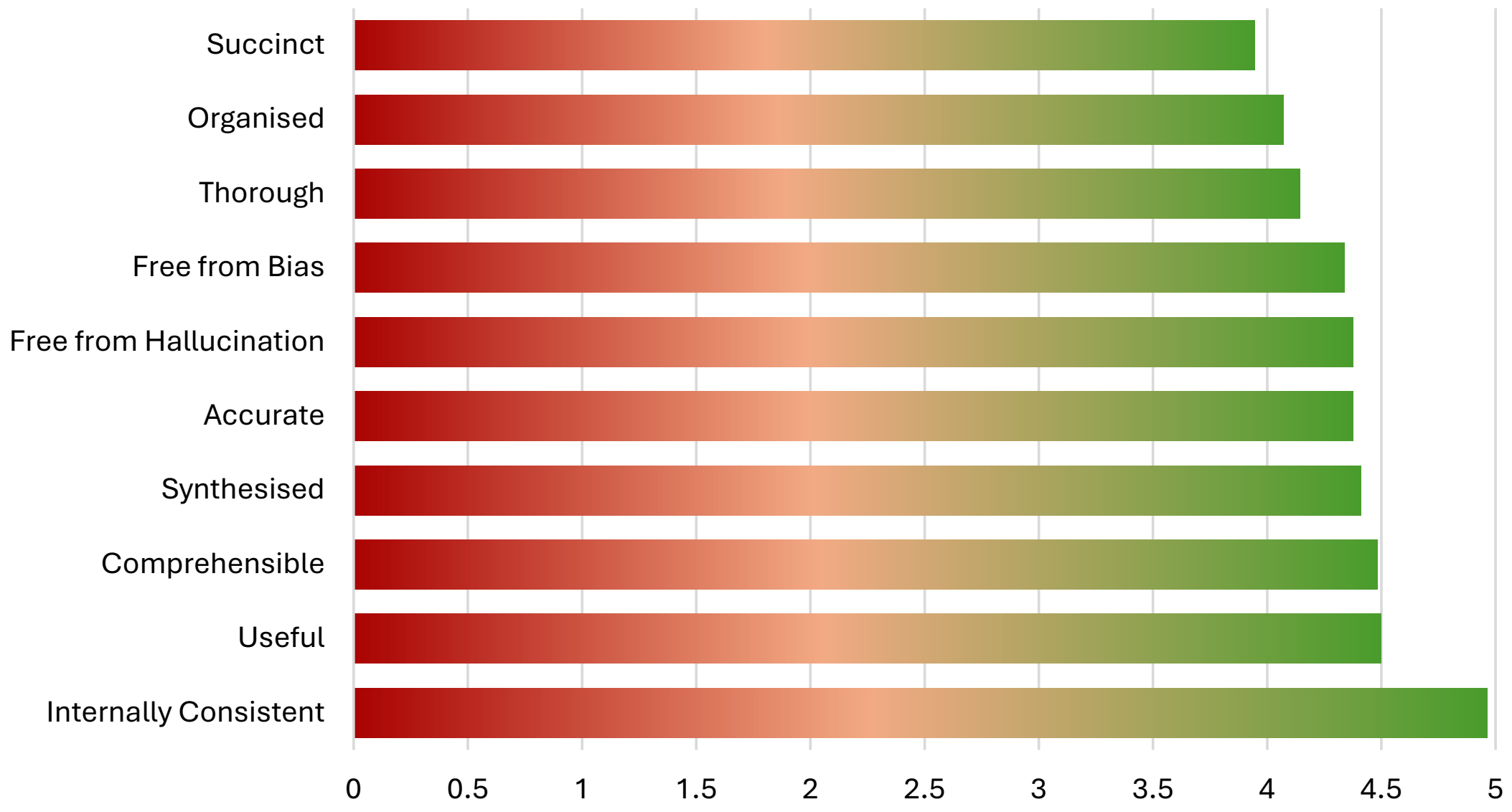

Figure S7: *Average PDQI-10 attribute scores across all CAIS products and scenarios. Scores reflect clinician ratings of ten documentation quality domains, with higher scores indicating better performance. "Succinct", "Organised", and "Thorough" were the lowest scoring attributes overall.*

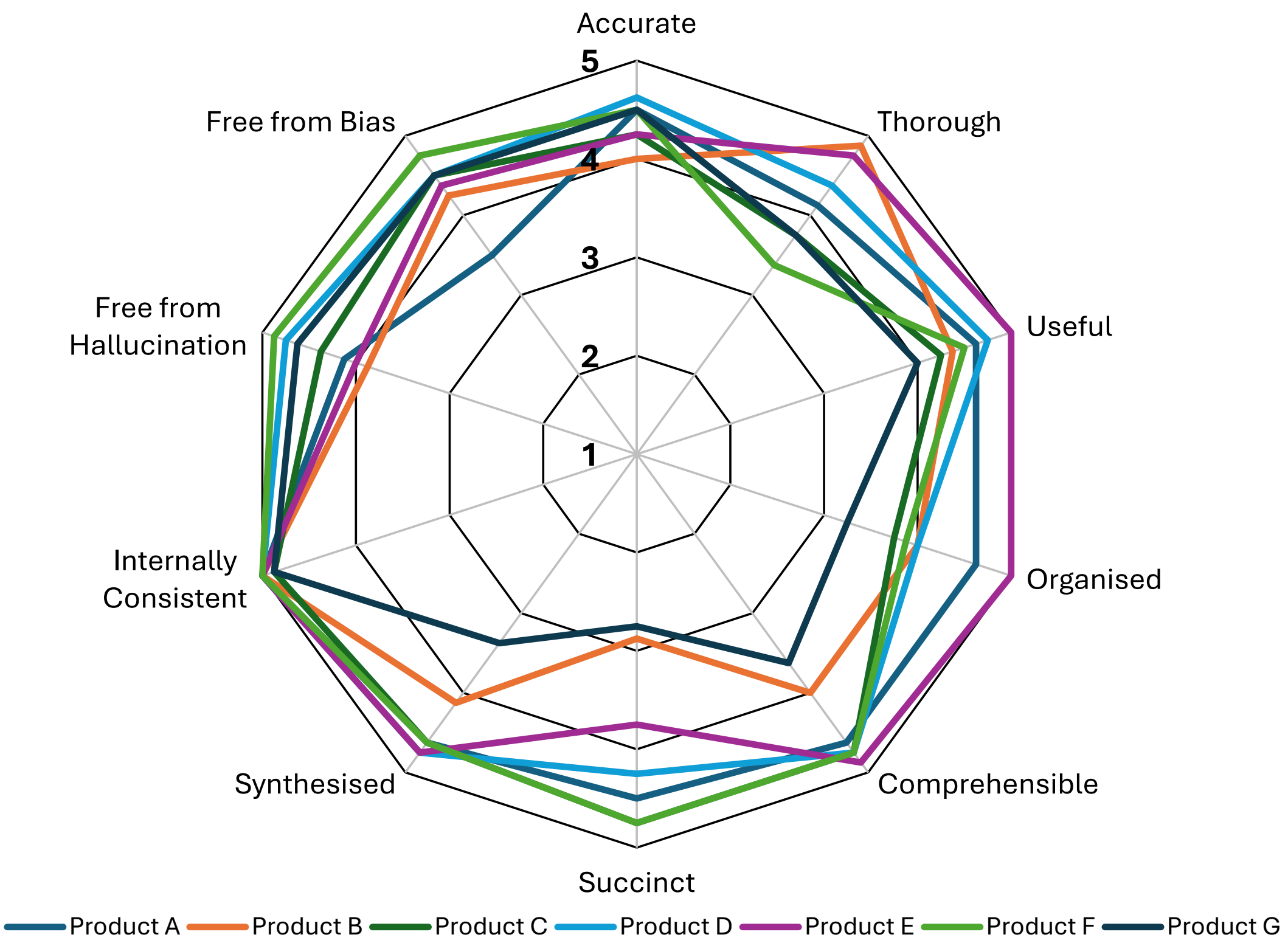

Figure S8: *A spider plot representing a breakdown of a CAIS's average PDQI-10 score, averaged across all eight clinical scenarios. It is clear that the 'succinct' and 'organised dimensions have weaker scores, whilst 'internally consistent' scores very highly.*

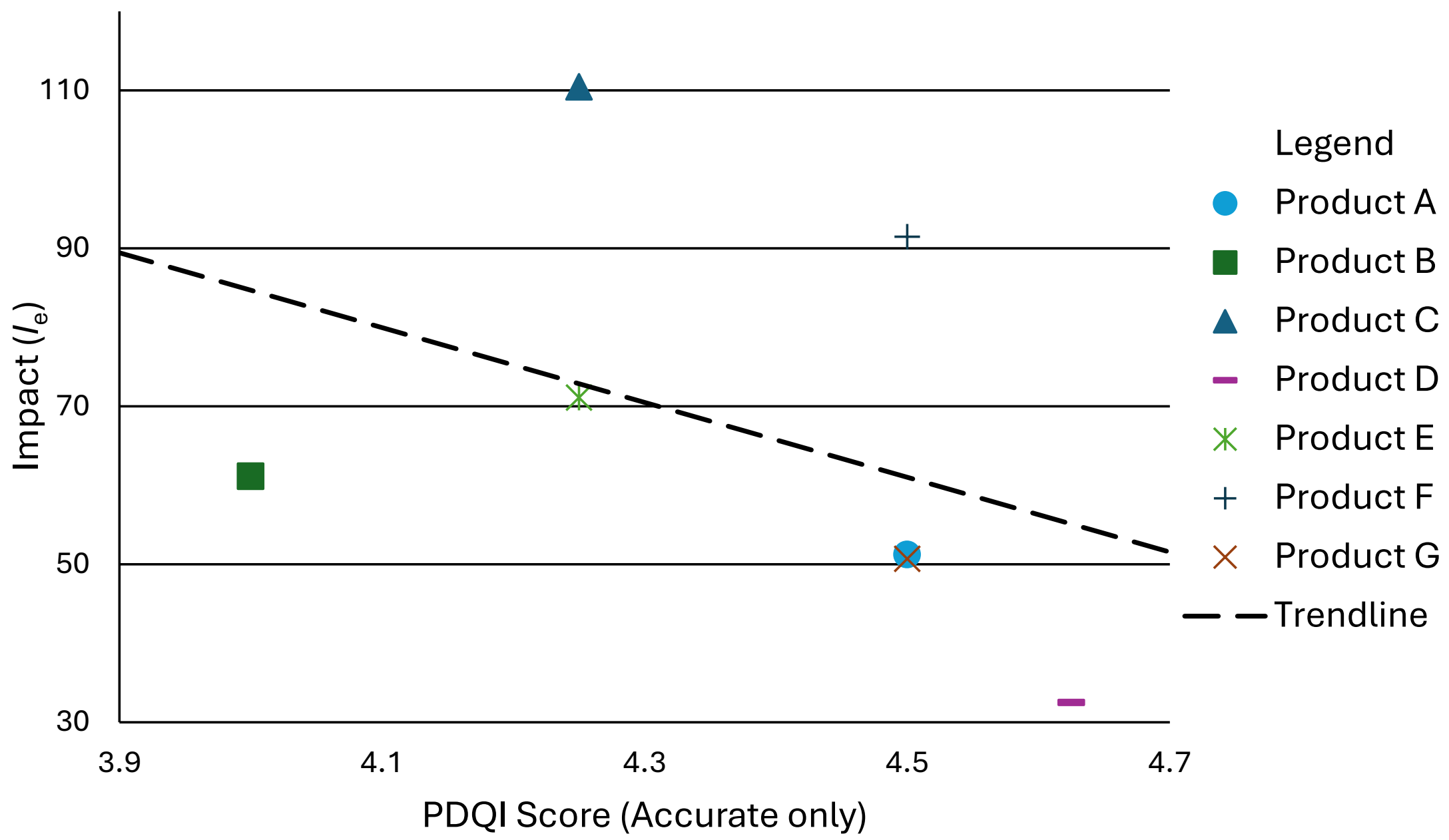

Figure S9: *A plot to explore the relationship between the exponential Impact Score for all eight scenarios and seven CAISs compared to the "Accurate" score on their PDQI-10.*

Tables

Table S1: *The Physician Documentation Quality Instrument system, published by Tierney et al. [4], and used in this work as a PDQI-10 scoring system. Each summary is given a mark between 1 and 5 for each attribute, which are then summed to give the score (maximum of 50).*

Attribute	Description of Ideal Note
Accurate	The note is true. It is free of incorrect information.
Thorough	The note is complete and free from omission and documents all of the issues of importance to the patient.
Useful	The note is extremely relevant, providing valuable information and/or analysis.
Organized	The note is well-formed and structured in a way that helps the reader understand the patient's clinical course.
Comprehensible	The note is clear, without ambiguity or sections that are difficult to understand.
Succinct	The note is brief, to the point, and without redundancy.
Synthesized	The note reflects the AI scribe's understanding of the patient's status and ability to develop a plan of care.
Internally Consistent	No part of the note ignores or contradicts any other part.
Free from Hallucination	The note is free of hallucination and only contains information verifiable by the transcript.
Free from Bias	The note is free of bias and contains only information verifiable by the transcript and not derived from characteristics of the patient or visit.

Table S2: *The average (interquartile range) result for various Clinical AI Scribe (CAIS) scores.*

**CAIS**	**Product A**	**Product B**	**Product C**	**Product D**	**Product E**	**Product F**	**Product G**
**Hallucinations** *(median)*	1 (1)	0 (0)	0 (0.75)	0 (0)	0 (1)	0 (0)	0 (0.75)
**Factual Inaccuracies** *(median)*	0 (0)	0 (1)	0 (0.75)	0 (1.75)	0 (0.75)	0.5 (1)	0 (1.75)
**Omissions** *(median)*	2.5 (2.5)	1.5 (2.75)	4 (5.25)	1 (3)	2 (0.75)	6 (1.75)	2.5 (3.25)
**Total Errors** *(median)*	3 (2.75)	2 (3.25)	5.5 (4.25)	2 (4)	2 (2.5)	6 (1.75)	3.5 (2.75)
**Word Count** *(median)*	158.5 (20.5)	256.5 (55.75)	129.5 (29.25)	274.5 (18.75)	192.5 (20)	182 (10.25)	378.5 (55.5)
**Impact Score (I_l)** *(median)*	8.63 (7.69)	6.25 (10.4)	15.8 (17.4)	5.5 (9.48)	7 (6.88)	17.4 (6.88)	8.17 (6.5)
**Impact Score (I_e)** *(median)*	50 (65.7)	40.4 (97.1)	102 (103)	25.1 (54.3)	44.4 (65.3)	113 (66.7)	47.1 (52.4)
**PDQI-10 score** *(mean)*	46 (7)	40 (6.5)	46 (4.25)	46.5 (4.25)	46 (4)	45.5 (4)	40.5 (7.5)

Table S3: *Error type by severity weight (w) across all tested CAISs. Severity weight ≥4 includes scores rated as "very relevant" (4) or "fatal" (5). Percentages rounded to 1 decimal place. This is visualised in figure 3 of the main paper.*

**Error Type**	**Total Errors**	**% of Total Errors**	**Errors with severity weight ≥4**	**% of Type Rated ≥4**	**% of All Severity ≥4 Errors**
Hallucinations	16	7.8%	3	18.8%	20.0%
Factual Inaccuracies	17	8.3%	3	17.6%	20.0%
Omission	171	83.8%	9	5.3%	60.0%
**Total**	204	100%	15	-	100%

Draper TC, *et al. BMJ Digit Health* 2025; 1:e000092. doi: 10.1136/bmjdhai-2025-000092

Table S4: *Representative examples of recurring errors across multiple CAISs. Examples were selected based on recurrence and clinical relevance (severity weight, w).*

Error Type	Specifics	# of CAISs (out of 7)	Mean Severity Weight (*w*)
Hallucination	Assumes and records suspected benign prostatic hyperplasia	2	3.8
Hallucination	Claimed NKA (no known allergies). But no allergies were discussed throughout consultation	1	4.2
Hallucination	Records the abdomen exam as "tender epigastrium". But no findings were actually vocalised.	1	4.2
Factual Inaccuracy	Records blood pressure as "Low". Whereas the blood pressure is *dangerously* low.	2	4.5
Factual Inaccuracy	Wrongly claims a 12 week wait for a opticians appointment, rather than the nighttime observation study	2	3.4
Factual Inaccuracy	Records that patient smokes occasional pipe. Actually is a non-smoker	2	2.6
Omission	Records difficulty producing stream, but not that sometimes completely unable to produce	6	3.2
Omission	Fails to note effectiveness of current pain medication for previously diagnosed carpel tunnel syndrome	6	2.4
Omission	Fails to record that dosage adjustment may be required, depending on test results	4	4.0
Omission	Fails to note patients concern about dementia	4	3.2
Omission	Fails to note patient takes OTC medication for gastric reflux	4	3.0
Omission	Fails to note that work is causing stress	4	2.4

Clinical Scenarios

**Scenario**	Diarrhoea
**Personal Details**	**Date of Birth:** 33 years old<br>**Sex:** F<br>**Occupation:** Teaching Assistant
**Presenting Complaint**	**Opening Statement:** “I've been getting some diarrhoea, for two or three weeks”<br><br>**Main Symptoms:** Frequent bowel movements (up to 8 times/day), blood in stools for the past few days, Crampy abdominal pain, primarily before bowel movements, Night-time bowel movements causing sleep disruption<br><br>**If asked specifically:** Weight loss over the past few weeks (clothes fitting looser)
**Medical History**	**Chronic illnesses:** None<br>**Operations:** Appendectomy at age 15<br>**Hospital Admissions:** None
**Drug History**	**Prescribed:** Contraceptive pill<br>**Over the Counter:** Paracetamol for headaches<br>**Allergies:** Amoxicillin (penicillin) – causes rash
**Family History**	**Illnesses in close relatives:** Aunt has tummy symptoms, don’t know the cause but its not cancer
**Social History**	**Home:** Lives with partner, Sam<br>**Effect on Life:** Cannot go to job because of 48h rule (must be clear of symptoms)<br>**Smoking:** In past, 10 cigarettes/day for 3 years (in college)<br>**Alcohol:** Bottle of wine and some shots at weekends only<br>**Recreational Drugs:** No<br>**Diet:** Normal<br>**Exercise:** None<br>**Recent Travel:** None recent, went to South Africa 3 years ago
**Ideas, Concerns, Expectations**	**Ideas (about cause of symptoms):** Maybe infection?<br>**Concerns:** Spreading infection to others, Sister’s friend has bowel cancer at only 30 years old, worried about a Colonoscopy<br>**Expectations:** Further testing or referral to specialist

**Scenario**	Sleep Apnoea
**Personal Details**	**Sex:** F<br>**Occupation:** Carer
**Presenting Complaint**	**Opening Statement:** "My husband said that I stop breathing during the night for about 20 seconds. When I wake up, I'm very tired."<br><br>**Symptoms:** Problems for about a year. Sleepy during the day, tired all the time, fall asleep in the afternoons<br><br>**If asked specifically:** Numbness and swelling of hands and feet, headaches, deteriorating eyesight, increased weight
**Medical History**	**Chronic illnesses:** Bilateral Carpal tunnel, wears wrist splints and use TENS machine for pain relief<br>**Operations:** None<br>**Hospital Admissions:** None
**Drug History**	**Prescribed:** Amitriptyline for headaches, gabapentin for carpal tunnel<br>**Over the Counter:** None<br>**Allergies:** None
**Family History**	**Illnesses in close relatives:** None
**Social History**	**Home:** Husband and 2 children<br>**Effect on Life:** Difficult to work shifts<br>**Smoking:** No<br>**Alcohol:** Special occasions only<br>**Recreational Drugs:** None<br>**Diet:** Normal<br>**Exercise:** None<br>**Recent Travel:** None
**Ideas, Concerns, Expectations**	**Ideas (about cause of symptoms):** None<br>**Concerns:** None<br>**Expectations:** None

Draper TC, *et al. BMJ Digit Health* 2025; 1:e000092. doi: 10.1136/bmjdhai-2025-000092

**Scenario**	Prostate
**Personal Details**	**Sex:** M<br>**Occupation:** Computer Programmer
**Presenting Complaint**	**Opening Statement:** "I think I might have one of those middle-aged problems, doctor"<br><br>**Symptoms:** Frequent needing a wee, urgent to go but then doesn't come out, getting up for a wee overnight, for a few months
**Medical History**	**Chronic illnesses:** None<br>**Operations:** None<br>**Hospital Admissions:** None
**Drug History**	**Prescribed:** None<br>**Over the Counter:** None<br>**Allergies:** None
**Family History**	**Illnesses in close relatives:** None
**Social History**	**Home:** Lives with wife<br>**Effect on Life:** Disturbed sleep and many breaks at work<br>**Smoking:** None<br>**Alcohol:** Beer every night<br>**Recreational Drugs:** None<br>**Diet:** Drinks lots of coffee and tea<br>**Exercise:** Moderate<br>**Recent Travel:** Only Europe
**Ideas, Concerns, Expectations**	**Ideas (about cause of symptoms):**<br>**Concerns:** Worried about Prostate cancer<br>**Expectations:** Urine tests, prostate exam?

Draper TC, *et al. BMJ Digit Health* 2025; 1:e000092. doi: 10.1136/bmjdhai-2025-000092

**Scenario**	Panic Attack
**Personal Details**	**Sex:** M<br>**Occupation:** University Lecturer
**Presenting Complaint**	**Opening Statement:** "Last night, I had a panic attack. I was at home watching telly and it just came on"<br>**Symptoms:** feeling anxious, clammy, short of breath, heart palpitations<br>**If asked specifically:** Stress at work, feeling isolated, high workload, house renovations
**Medical History**	**Chronic illnesses:** None<br>**Operations:** None<br>**Hospital Admissions:** None
**Drug History**	**Prescribed:** lansoprazole for acid reflux.<br>**Over the Counter:** None<br>**Allergies:** None
**Family History**	**Illnesses in close relatives:** None
**Social History**	**Home:** Wife and two daughters.<br>**Effect on Life:** Worried about complaints at work<br>**Smoking:** None<br>**Alcohol:** Wine or maybe a beer at the weekend.<br>**Recreational Drugs:** None<br>**Diet:** Normal<br>**Exercise:** Moderate<br>**Recent Travel:** None
**Ideas, Concerns, Expectations**	**Ideas (about cause of symptoms):** Panic attack?<br>**Concerns:** Worried about effect on work<br>**Expectations:** Beta blockers

Draper TC, *et al. BMJ Digit Health* 2025; 1:e000092. doi: 10.1136/bmjdhai-2025-000092

**Scenario**	Abdominal Pain
**Personal Details**	**Sex:** F<br>**Occupation:** Work from home Web Designer
**Presenting Complaint**	**Opening Statement:** "I've been having really bad tummy pain and last night it got really bad"<br><br>**Symptoms:** Pain top of abdomen through to the back, keeping awake at night<br><br>**If asked specifically:** Been sick 3 times today, blood in stools
**Medical History**	**Chronic illnesses:** None<br>**Operations:** None<br>**Hospital Admissions:** None
**Drug History**	**Prescribed:** None<br>**Over the Counter:** None<br>**Allergies:** None
**Family History**	**Illnesses in close relatives:** None
**Social History**	**Home:** Live alone except for dog, Scraps<br>**Effect on Life:** Pain is a distraction when trying to work<br>**Smoking:** Occasional small cigar<br>**Alcohol:** Drink a bottle of red wine every day at lunchtime, more drinks in the evening at the Golf Club<br>**Recreational Drugs:** None<br>**Diet:** Normal<br>**Exercise:** Moderate<br>**Recent Travel:** None
**Ideas, Concerns, Expectations**	**Ideas (about cause of symptoms):** Might be a stomach ulcer?<br>**Concerns:** Can't have wine in hospital<br>**Expectations:** Not serious illness

**Scenario**	Headache
**Personal Details**	**Sex:** M<br>**Occupation:** work in IT and recruitment. Lots of talking on the phone.
**Presenting Complaint**	**Opening Statement:** “I've got this terrible headache that feels like a band around my head”<br><br>**Symptoms:** for 4-6 weeks, pain gets worse during the day<br><br>**If asked specifically:** Pain around the base of the neck and into the shoulders, feeling really tight.
**Medical History**	**Chronic illnesses:** None<br>**Operations:** None<br>**Hospital Admissions:** None
**Drug History**	**Prescribed:** None<br>**Over the Counter:** ibuprofen gave me indigestion, and the paracetamol made me constipated. Neither of them got rid of the headache.<br>**Allergies:** None
**Family History**	**Illnesses in close relatives:** None
**Social History**	**Home:** Wife and 2 kids, 10 and 12.<br>**Effect on Life:**<br>**Smoking:** No<br>**Alcohol:** occasional 1-2 drinks<br>**Recreational Drugs:** None<br>**Diet:** Normal<br>**Exercise:** Moderate<br>**Recent Travel:** None
**Ideas, Concerns, Expectations**	**Ideas (about cause of symptoms):** Is it brain cancer?<br>**Concerns:** Is it something serious?<br>**Expectations:**

**Scenario**	Skin Rash
**Personal Details**	**Sex:** M<br>**Occupation:** Retired telephone engineer
**Presenting Complaint**	**Opening Statement:** “I have a rash, and I think it is getting worse”<br><br>**Symptoms:** Rash started on tummy 2-3 days ago, now spreading onto arms chest and back, not itch or painful<br><br>**If asked specifically:** Nosebleed the last couple of days, also blood in mouth
**Medical History**	**Chronic illnesses:** Atrial fibrillation<br>**Operations:** None<br>**Hospital Admissions:** None
**Drug History**	**Prescribed:** Apixaban (blood thinning medication to prevent clots)<br>**Over the Counter:** None<br>**Allergies:** None
**Family History**	**Illnesses in close relatives:** No
**Social History**	**Home:** Partner with dementia, no family nearby, no pets<br>**Effect on Life:** No hobbies, don’t get out much<br>**Smoking:** three pipes a week<br>**Alcohol:** a few pints of beer a week<br>**Recreational Drugs:** None<br>**Diet:** Normal<br>**Exercise:** None<br>**Recent Travel:** None
**Ideas, Concerns, Expectations**	**Ideas (about cause of symptoms):** No<br>**Concerns:** Worried about heart problem, wife<br>**Expectations:** No

Draper TC, *et al. BMJ Digit Health* 2025; 1:e000092. doi: 10.1136/bmjdhai-2025-000092

**Scenario**	Memory Loss
**Personal Details**	**Sex:** F<br>**Occupation:** Events organiser.
**Presenting Complaint**	**Opening Statement:** “I have been having some memory issues. Just struggling to remember the basic things”<br><br>**Symptoms:** Poor memory for about a year, having to write notes<br><br>**If asked specifically:** Poor sleep, night sweats
**Medical History**	**Chronic illnesses:** None<br>**Operations:** None<br>**Hospital Admissions:** None
**Drug History**	**Prescribed:** None<br>**Over the Counter:** Red Clover supplements for menopause symptoms<br>**Allergies:** None
**Family History**	**Illnesses in close relatives:** No
**Social History**	**Home:** Cheating husband, adult children moved out<br>**Effect on Life:** anxious and scared, people are starting to notice<br>**Smoking:** Two to three glasses a week<br>**Alcohol:** No<br>**Recreational Drugs:** None<br>**Diet:** Normal<br>**Exercise:** Some<br>**Recent Travel:** None
**Ideas, Concerns, Expectations**	**Ideas (about cause of symptoms):** dementia<br>**Concerns:** Worried about it getting worse<br>**Expectations:** No

Draper TC, *et al. BMJ Digit Health* 2025; 1:e000092. doi: 10.1136/bmjdhai-2025-000092

References

1 R Core Team. _R: A Language and Environment for Statistical Computing_. 2024.

2 Posit Team. RStudio: Integrated Development Environment for R. 2024.

3 IBM Corp. SPSS Statistics for Windows (Version 29). 2022.

4 Tierney AA, Gayre G, Hoberman B, *et al.* Ambient Artificial Intelligence Scribes to Alleviate the Burden of Clinical Documentation. *NEJM Catal*. 2024;5:1–15. doi: 10.1056/CAT.23.0404
